# Global burden of anemia attributed to chronic kidney disease: prevalence, years lived with disability, and predictions to 2035 (Global burden of disease 2021)

**DOI:** 10.3389/fnut.2025.1690686

**Published:** 2025-11-18

**Authors:** Wenshuai Zheng, Huaxin Chen, Wentao Zhang, Yibo Cai, Zongze Wu, Lixun Guan

**Affiliations:** 1 Department of Hematology, Hainan Hospital of Chinese PLA General Hospital, Sanya, Hainan, China; 2 Department of Anesthesiology, Hainan Hospital of Chinese PLA General Hospital, Sanya, Hainan, China; 3 Department of Pediatrics, Hainan Hospital of Chinese PLA General Hospital, Sanya, Hainan, China; 4 Department of Osteology, Hainan Hospital of Chinese PLA General Hospital, Sanya, Hainan, China; 5 Department of Rehabilitation Medicine, Hainan Hospital of Chinese PLA General Hospital, Sanya, Hainan, China

**Keywords:** anemia burden, chronic kidney disease, epidemiology, global burden of disease study, prevalence

## Abstract

**Background:**

As a common complication of chronic kidney disease (CKD), anemia is associated with increased mortality and reduced quality of life. Despite its severe impact, there is a lack of high-quality data on the global burden of anemia attributed to CKD. This study aims to provide a comprehensive analysis of the anemia burden attributed to CKD.

**Methods:**

Using data from the Global Burden of Disease study (GBD) 2021, we report the prevalence and years lived with disability (YLDs) of anemia attributed to CKD across different sexes, ages, and regions; assess the association between anemia burden attributed to CKD and the socio-demographic index (SDI); and quantify and predict temporal trends of anemia burden attributed to CKD.

**Results:**

In 2021, there were 63.75 million (95% uncertainty interval [UI]: 59.05 to 68.37) cases and 1.70 million (95% UI: 1.13 to 2.43) YLDs of anemia attributed to CKD globally. Compared with 1990, the prevalence and YLDs increased by 96.24 and 74.78%, respectively, which was largely driven by population growth and aging. The global age-standardized prevalence rate (ASPR) and YLD rate per 100,000 were 762.12 (95% UI: 707.32 to 817.37) and 20.34 (95% UI: 13.54 to 29.09) in 2021, which decreased by 9.39 and 18.93% in comparison with those in 1990. However, the decline in ASPR stagnated after 2010, with a slight increase observed between 2010 and 2015. A negative relationship between SDI and the anemia burden attributed to CKD was observed at both regional and national levels. Women had higher ASPR and age-standardized YLD rates compared to men, and the burden attributed to CKD increased with age. Predictive analysis indicated that the prevalence of cases will continue to rise, while the YLDs, ASPR, and age-standardized YLD rates are expected to decline consistently.

**Conclusion:**

Anemia attributed to CKD is a major public health issue across the world, with persistent regional and socioeconomic disparities. Continued efforts, including addressing socioeconomic disparities, improving access to healthcare, and innovative treatments, are essential to reduce the anemia burden attributed to CKD.

## Introduction

Chronic kidney disease (CKD) represents a growing global public health challenge, imposing a substantial economic burden on both affected families and healthcare systems worldwide. Approximately 10% of adults are affected by CKD globally (1), with a prevalence exceeding 40% among individuals aged over 60 (2, 3).

Anemia is a frequent complication of CKD that typically arises from reduced erythropoietin production, impaired iron metabolism, nutritional deficits, and oxidative stress (4). Anemia attributed to CKD is associated with numerous adverse outcomes, including increased morbidity and mortality, as well as reduced quality of life (5–8). A Swedish cohort study involving 14,415 CKD patients reported that anemia was associated with a 2.5-fold higher risk of major adverse cardiovascular events (9). Previous studies on the anemia burden attributed to CKD have revealed a high and variable prevalence, ranging from 15.4 to 91.9% across different regions (10–14). This high prevalence represents a significant clinical and economic concern, as patients with both CKD and anemia utilize more healthcare resources and incur higher costs compared to those without anemia (15–17). Economic analyses underscore the considerable costs of anemia management, particularly among dialysis-dependent patients, with annual expenditures exceeding US$76.8 billion in the United States alone (18).

Early screening and optimal management of anemia attributed to CKD have been shown to reduce morbidity and mortality and improve patients’ quality of life (7, 19), particularly considering the fact that the incidence and mortality of CKD increased consistently (20, 21). However, high-quality, internally consistent, and comprehensive estimates of the anemia burden attributed to CKD are lacking, limiting the development of targeted intervention strategies. Previous evaluations have often addressed anemia attributed to CKD within the broader context of anemia burden or in specific regions (22–24). The Global Burden of Disease (GBD) Study 2021 provides a reliable framework for assessing the current prevalence and years lived with disability (YLDs) of anemia attributed to CKD (25). Although previous studies using GBD assessed the anemia burden for all causes, they did not specifically estimate the anemia burden attributed to CKD (22, 23). Therefore, this study aims to describe the global anemia burden attributed to CKD, including prevalence and YLDs, with emphasis on age, gender, and regional distributions, as well as temporal trends from 1990 to 2021, which have been rarely described in previous studies. These findings would inform specific interventions, including treatment planning, resource allocation, and the development of prevention strategies.

## Methods

### Data source

We conducted a secondary analysis of the GBD 2021 study.^1^ The detailed methods used for the GBD 2021 study have been described elsewhere (25, 26). In brief, the 2021 GBD study includes nationally representative surveys, censuses, and meta-analysis results and provides an accessible epidemiological assessment of 371 diseases, injuries, and impairments, as well as 88 risk factors, encompassing 204 nations and territories, utilizing the most recent epidemiological data and enhanced standardized methodologies. The GBD 2021 utilized data from a variety of sources, including peer-reviewed literature, survey data, disease registers, and hospital inpatient records, to ensure the quality and comprehensiveness of the anemia-related analysis. The data were then synthesized with the Cause of Death Ensemble model, spatiotemporal Gaussian process regression, and a Bayesian meta-regression modeling tool (DisMod-MR 2.1). The GBD study provides a standardized methodology for estimating the determinants and their 95% uncertainty interval (UI) of prevalence and YLDs in terms of absolute number, crude rate per 100,000 population, and age-standardized rate.

This study utilized the sociodemographic index (SDI)—a composite indicator based on estimates of total fertility rate in those younger than 25 years, mean years of education in individuals older than 15 years, and lag-distributed income per capita (27),—to explore the relationship between the anemia burden among WRA and the development status of a region or country. The SDI ranges from 0 (less developed) to 1 (most developed). The 204 countries and territories were classified into five SDI regions based on SDI quintiles in 2021: low, low-middle, middle, high-middle, and high.

This study adhered to the Guidelines for Accurate and Transparent Health Estimates Reporting (28). The study did not involve any personal or sensitive information. Consequently, no ethical approval was required for the execution of this study.

### Disease definitions

Anemia is defined by reduced concentrations of hemoglobin (Hb) in the blood, irrespective of etiology, red blood cell morphology, or function. Anemia diagnosis follows the WHO hemoglobin thresholds (g/L) (29), including < 110 g/L for children aged 6–59 months and pregnant women, < 115 g/L for children aged 5–11 years of age, < 120 g/L for children aged 12–14 years and non-pregnant women (15 years of age and above), and < 130 g/L for men (15 years of age and above). CKD is defined as kidney injury or impaired renal function that persists for 3 months or longer, regardless of the cause (30). In the GBD 2021, the prevalence of anemia was estimated by generating counterfactual distributions based on age- and sex-specific prevalence of anemia-causing conditions and assessing their impact on blood hemoglobin concentration (25). The age- and sex-specific anemia prevalence for CKD was analyzed as part of the overall anemia causal attribution.

### Statistical analysis

To identify the direction and magnitude of changes in the prevalence and YLDs of anemia attributed to CKD over time, we calculated the annual percentage change (APC) and the average APC (AAPC) and the corresponding 95% confidence interval (CI) with joinpoint regression analysis. A positive APC or AAPC indicates an increase, while a negative value indicates a decrease. In order to identify the year with the most marked changes, we detected trends in the data over time and fitted the optimal model curve to the data by segmenting the data into multiple linear pieces on a logarithmic graph using the joinpoint regression analysis. These segmented points are referred to as “joinpoints.” The “joinpoints” were data-driven, identified based on statistical significance. In detail, in the joinpoint regression analysis, a logarithmic linear model (ln y = *β* *x) is used for segmented regression, and the grid search method (GSM) is used to establish all possible connection points. The mean squared errors (MSEs) corresponding to each possible scenario are calculated, and the grid point with the smallest MSE is selected as the connection point. Then, the establishment of the optimal model for the regression of connection points (i.e., the number of connection points) is determined using the Monte Carlo permutation test. We set the maximum number of potential connection points to 4 and the minimum number of potential connection points to 0 (26, 31).

To examine potential non-linear associations between anemia burden attributed to CKD and SDI, we utilized Pearson correlation analysis to estimate the strength and direction of correlations between SDI and age-standardized YLD rates. This approach helped identify nations where anemia prevalence and YLDs substantially diverged from expectations based on socioeconomic status. Such a comprehensive analysis clarifies how socioeconomic factors influence the anemia burden attributed to CKD.

The Bayesian age-period-cohort (BAPC) model was used to predict the prevalence, number of YLDs, and the rate of anemia attributed to CKD from 2022 to 2035. In brief, the age-period-cohort model, a logarithmic linear Poisson model, postulates the multiplicative impact of age, period, and cohort, all following a Poisson distribution and utilizing a link function specific to the model. The BAPC model is expressed as n_ij_ = log(λ_ij_) = *μ* + α_i_ + β_j_ + γ_k_, where λ_ij_ denotes the count of cases, μ denotes the intercept, and α_i_, β_j_, and γ_k_ signify the effect of age, period, and cohort, respectively (32, 33).

A robust decomposition method was used for the data analysis. This method attributes differences in prevalence and YLDs between two time points to changes in three independent factors: (a) the age structure of the population, the shifting of which toward greater numbers of older individuals are referred to as population aging, (b) population size, and (c) age-specific rate, which reflects the joint effect of all factors other than age structure and population size. In this method, the three two-way interactions are equally divided into relevant factors. Detailed derivations have been described in previous studies (34).

All analyses and visualizations were conducted in R version 4.3.3 (https://www.r-project.org) and JD_GBDR (V2.24, Jingdong Medical Technology Co., Ltd.). All statistical tests were two-sided, and *p*-values of < 0.05 were considered statistically significant.

## Results

### Global level

**Figure 1:**
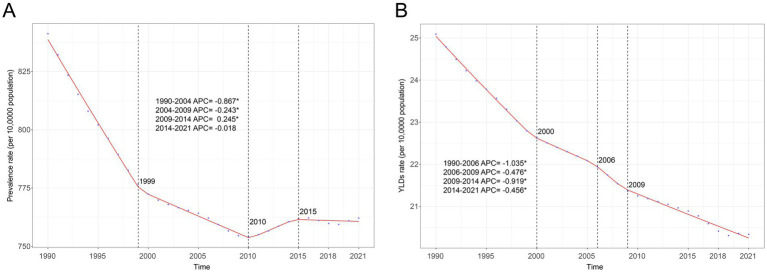
Joinpoint regression analysis of global anemia attributed to CKD. **(A)** Age-standardized prevalence rate per 100,000 from 1990 to 2021 and **(B)** age-standardized YLD rate per 100,000 from 1990 to 2021. APC, annual percentage change; CKD, chronic kidney disease; YLDs, years lived with disability.

In 2021, there were 63.75 million (95% UI: 59.05 to 68.37) prevalence cases and 1.70 million (95% UI: 1.13 to 2.43) YLDs in anemia attributed to CKD across the globe. Compared to 1990, prevalence cases and YLDs in 2021 increased by 96.24 and 74.78%, respectively. However, the global age-standardized prevalence rate (ASPR) and age-standardized YLD rate of anemia attributed to CKD have decreased over the past 30 years. The ASPR per 100,000 population decreased from 841.18 (95% UI: 783.12 to 906.48) in 1990 to 762.12 (95% UI: 707.32 to 817.37) in 2021, with an AAPC of −0.317 (95% CI: −0.323 to −0.312) (Table 1; Figure 1A). In the same period, the age-standardized YLD rate per 100,000 population decreased from 25.09 (95% UI: 16.78 to 35.65) in 1990 to 20.34 (95% UI: 13.54 to 29.09) in 2021, with an AAPC of −0.673 (95% CI: −0.681 to −0.663) (Table 1; Figure 1B). As shown in Figure 1, the most significant decline in global ASPR occurred between 1990 and 1999 [APC = −0.867 (95% CI: −0.897 to −0.837)]. However, the decreased trend of ASPR stagnated after 2010, and there was a rising trend between 2010 and 2015 [APC = 0.245 (95% CI: 0.121 to 0.369)], which primarily contributed to the increase of ASPR in the high SDI (Supplementary Figure S1).

**Table 1:** Prevalence and YLDs of anemia attributed to CKD, and their temporal trends from 1990 to 2021 at the global and regional levels.

Location	1990		2021		1990–2021	1990		2021		1990–2021
	Prevalence cases (95% UI)	Prevalence rate per 100,000 (95% UI)	Prevalence cases (95% UI)	Prevalence rate per 100,000 (95% UI)	AAPC (%), (95% CI)	YLDs (95% UI)	YLD rate per 100,000 (95% UI)	YLDs (95% UI)	YLD rate per 100,000 (95% UI)	AAPC (%), (95% CI)
Global	32,486,224 (30,356,876 to 35,047,084)	841.18 (783.12 to 906.48)	63,751,624 (59,045,051 to 68,372,650)	762.12 (707.32 to 817.37)	−0.317 (−0.323 to −0.312)	972,375 (650,009 to 1,385,335)	25.09 (16.78 to 35.65)	1,699,516 (1,131,250 to 2,433,689)	20.34 (13.54 to 29.09)	−0.673 (−0.681 to −0.663)
Male	14,346,016 (13,323,901 to 15,630,185)	839.10 (780.83 to 910.92)	28,208,709 (26,097,779 to 30,409,324)	748.10 (691.69 to 807.21)	−0.374 (−0.379 to −0.368)	363,831 (242,840 to 518,598)	22.38 (14.95 to 31.57)	580,117 (379,681 to 841,698)	15.81 (10.36 to 22.71)	−1.116 (−1.124 to −1.109)
Female	18,140,208 (16,913,628 to 19,524,601)	854.93 (798.01 to 921.56)	35,542,915 (32,942,946 to 38,267,108)	784.53 (728.64 to 842.42)	−0.275 (−0.283 to −0.268)	608,543 (407,384 to 867,082)	28.29 (18.89 to 40.31)	1,119,399 (746,450 to 1,608,623)	24.83 (16.56 to 35.73)	−0.408 (−0.419 to −0.399)
High SDI	6,831,434 (6,294,908 to 7,497,741)	631.20 (583.76 to 687.86)	12,937,734 (11,594,440 to 14,338,944)	575.32 (517.79 to 636.52)	−0.295 (−0.306 to −0.285)	116,697 (73,289 to 172,849)	10.86 (6.86 to 16.08)	219,456 (138,759 to 328,837)	9.48 (5.96 to 14.33)	−0.435 (−0.443 to −0.428)
High-middle SDI	6,546,839 (6,049,891 to 7,127,759)	714.28 (658.63 to 777.30)	9,876,782 (9,030,632 to 10,648,516)	526.27 (483.55 to 565.57)	−0.981 (−0.989 to −0.974)	161,892 (105,819 to 233,309)	17.96 (11.79 to 25.63)	212,507 (136,908 to 309,323)	11.37 (7.33 to 16.54)	−1.459 (−1.473 to −1.446)
Middle SDI	9,868,168 (9,081,277 to 10,734,606)	958.14 (883.95 to 1046.64)	20,291,028 (18,699,076 to 21,983,141)	798.65 (737.73 to 861.88)	−0.58 (−0.59 to −0.571)	301,289 (201,303 to 429,323)	30.69 (20.54 to 43.39)	535,887 (353,697 to 770,788)	21.55 (14.24 to 30.81)	−1.127 (−1.138 to −1.116)
Low-middle SDI	6,735,513 (6,240,338 to 7,312,831)	1077.42 (996.26 to 1174.43)	15,019,551 (13,936,960 to 16,277,999)	1050.06 (971.42 to 1140.28)	−0.083 (−0.094 to −0.073)	284,512 (192,875 to 401,690)	46.64 (31.74 to 65.62)	528,032 (357,456 to 744,046)	38.05 (25.87 to 53.82)	−0.652 (−0.664 to −0.641)
Low SDI	2,472,885 (2,274,376 to 2,695,336)	1016.33 (931.79 to 1110.53)	5,573,562 (5,150,095 to 6,019,435)	995.84 (916.54 to 1080.20)	−0.07 (−0.076 to −0.064)	107,237 (72,771 to 149,542)	45.82 (31.06 to 63.05)	202,492 (136,829 to 287,497)	38.72 (26.28 to 54.37)	−0.542 (−0.55 to −0.534)
Andean Latin America	132,454 (119,249 to 147,457)	569.78 (507.17 to 640.82)	242,931 (216,487 to 272,859)	409.48 (362.36 to 461.34)	−1.061 (−1.073 to −1.051)	3,198 (2049 to 4,677)	13.82 (8.88 to 19.94)	4,878 (3,097 to 7,265)	8.24 (5.23 to 12.23)	−1.65 (−1.66 to −1.64)
Australasia	109,319 (92,469 to 131,869)	492.68 (418.17 to 591.29)	249,955 (206,209 to 306,611)	414.66 (346.56 to 504.35)	−0.575 (−0.64 to −0.536)	1906 (1,207 to 2,847)	8.75 (5.53 to 12.88)	4,173 (2,526 to 6,419)	6.85 (4.18 to 10.50)	−0.809 (−0.872 to −0.765)
Caribbean	200,558 (179,692 to 225,735)	770.39 (682.92 to 867.64)	401,404 (356,259 to 443,422)	748.32 (665.25 to 826.02)	−0.097 (−0.102 to −0.093)	4,374 (2,877 to 6,513)	17.14 (11.33 to 25.62)	8,176 (5,285 to 12,480)	15.12 (9.77 to 22.90)	−0.405 (−0.411 to −0.399)
Central Asia	753,089 (682,295 to 848,101)	1580.52 (1428.16 to 1779.32)	1,280,182 (1,151,960 to 1,434,237)	1615.07 (1455.29 to 1800.63)	0.066 (0.061 to 0.071)	24,083 (15,983 to 35,378)	49.41 (32.85 to 72.23)	36,107 (23,378 to 53,926)	43.68 (28.66 to 64.84)	−0.4 (−0.409 to −0.392)
Central Europe	903,346 (818,831 to 994,151)	677.19 (617.68 to 739.56)	1,214,132 (1,099,707 to 1,333,265)	603.57 (551.82 to 660.03)	−0.37 (−0.378 to −0.363)	21,561 (13,946 to 31,508)	16.39 (10.63 to 23.79)	25,270 (16,262 to 37,491)	12.21 (7.83 to 18.33)	−0.948 (−0.955 to −0.941)
Central Latin America	588,517 (546,592 to 641,158)	700.08 (643.27 to 768.31)	1,600,975 (1,476,233 to 1,736,995)	656.59 (604.70 to 711.45)	−0.205 (−0.21 to −0.2)	12,862 (8,328 to 18,875)	15.71 (10.18 to 22.77)	31,974 (20,712 to 47,500)	13.18 (8.54 to 19.54)	−0.571 (−0.578 to −0.563)
Central Sub-Saharan Africa	382,313 (328,260 to 443,207)	1573.69 (1363.26 to 1809.69)	925,957 (809,409 to 1,056,026)	1531.84 (1329.91 to 1743.46)	−0.091 (−0.102 to −0.08)	14,027 (9,389 to 20,298)	58.27 (39.62 to 82.80)	26,839 (17,650 to 39,070)	45.70 (29.87 to 65.92)	−0.784 (−0.794 to −0.774)
East Asia	5,879,239 (5,398,796 to 6,421,814)	716.66 (656.76 to 782.00)	8,538,579 (7,771,758 to 9,371,900)	431.26 (394.33 to 471.04)	−1.626 (−1.653 to −1.603)	176,064 (116,720 to 249,045)	23.83 (15.95 to 33.91)	202,914 (132,596 to 290,845)	10.61 (6.93 to 15.12)	−2.569 (−2.594 to −2.548)
Eastern Europe	2,029,244 (1,743,826 to 2,358,566)	802.18 (696.67 to 918.63)	2,453,293 (2,124,762 to 2,834,246)	719.56 (626.84 to 820.29)	−0.345 (−0.35 to −0.34)	45,404 (29,051 to 68,026)	18.25 (11.77 to 26.93)	49,613 (31,217 to 72,897)	14.71 (9.26 to 21.55)	−0.696 (−0.707 to −0.684)
Eastern Sub-Saharan Africa	451,657 (415,493 to 490,009)	542.32 (496.49 to 594.58)	981,576 (904,339 to 1,061,590)	514.99 (470.35 to 563.06)	−0.172 (−0.189 to −0.16)	15,536 (10,362 to 22,157)	18.79 (12.69 to 26.72)	27,264 (17,903 to 39,910)	14.72 (9.78 to 21.29)	−0.787 (−0.794 to −0.782)
High-income Asia Pacific	1,695,803 (1,479,538 to 1,944,644)	896.63 (786.34 to 1031.46)	3,673,315 (3,070,457 to 4,328,397)	660.18 (553.76 to 776.23)	−0.984 (−1.002 to −0.97)	24,298 (14,718 to 37,731)	13.73 (8.47 to 21.01)	62,594 (38,435 to 93,818)	9.95 (6.02 to 15.22)	−1.035 (−1.049 to −1.022)
High-income North America	2,135,480 (1,927,296 to 2,386,674)	595.36 (540.26 to 661.86)	4,539,513 (3,869,673 to 5,366,310)	678.80 (578.75 to 796.49)	0.425 (0.401 to 0.443)	35,452 (22,429 to 52,116)	9.80 (6.21 to 14.47)	73,173 (46,001 to 111,686)	10.86 (6.80 to 16.52)	0.339 (0.318 to 0.356)
North Africa and Middle East	1,667,561 (1,545,140 to 1,807,267)	905.38 (837.21 to 986.84)	3,549,996 (3,241,471 to 3,913,046)	787.49 (714.66 to 863.13)	−0.441 (−0.45 to −0.431)	46,618 (30,686 to 66,454)	25.79 (17.16 to 36.41)	87,592 (58,046 to 126,409)	19.59 (13.04 to 28.19)	−0.872 (−0.882 to −0.862)
Oceania	32,681 (29,085 to 36,696)	1062.49 (942.62 to 1195.30)	85,278 (75,472 to 96,636)	1114.25 (981.63 to 1253.29)	0.153 (0.147 to 0.157)	921 (594 to 1,342)	33.16 (22.04 to 47.06)	2,188 (1,432 to 3,266)	31.94 (20.89 to 47.52)	−0.121 (−0.129 to −0.115)
South Asia	6,924,406 (6,394,247 to 7,543,929)	1165.20 (1073.07 to 1273.73)	16,656,701 (15,357,588 to 18,058,350)	1125.96 (1038.89 to 1221.98)	−0.111 (−0.129 to −0.094)	335,078 (228,658 to 465,434)	58.91 (40.28 to 81.82)	655,301 (443,569 to 918,707)	46.47 (31.70 to 65.28)	−0.761 (−0.776 to −0.748)
Southeast Asia	3,073,915 (2,820,958 to 3,341,850)	1196.48 (1087.40 to 1318.58)	7,120,216 (6,502,555 to 7,795,828)	1172.36 (1067.22 to 1289.52)	−0.062 (−0.075 to −0.046)	81,832 (53,750 to 117,033)	34.08 (22.35 to 48.60)	166,644 (108,276 to 241,395)	28.79 (18.78 to 41.46)	−0.531 (−0.546 to −0.514)
Southern Latin America	219,055 (193,329 to 253,899)	490.00 (431.76 to 566.09)	351,189 (308,706 to 405,781)	402.33 (353.04 to 463.19)	−0.65 (−0.67 to −0.632)	3,845 (2,454 to 5,827)	9.18 (5.88 to 13.72)	6,411 (4,091 to 9,751)	7.14 (4.54 to 10.87)	−0.814 (−0.83 to −0.792)
Southern Sub-Saharan Africa	329,327 (290,231 to 378,457)	1176.42 (1025.96 to 1353.77)	626,863 (565,016 to 698,805)	1115.92 (1007.14 to 1249.47)	−0.162 (−0.176 to −0.144)	9,591 (6,228 to 14,114)	33.07 (21.67 to 48.50)	17,405 (11,342 to 25,197)	29.87 (19.50 to 43.35)	−0.319 (−0.335 to −0.302)
Tropical Latin America	970,336 (822,163 to 1,131,309)	1086.40 (921.29 to 1266.76)	2,100,626 (1,773,962 to 2,497,967)	836.38 (708.66 to 992.28)	−0.832 (−0.843 to −0.821)	22,703 (14,572 to 33,475)	25.29 (16.29 to 37.66)	44,582 (27,738 to 68,794)	17.77 (11.04 to 27.49)	−1.13 (−1.149 to −1.113)
Western Europe	2,775,555 (2,527,159 to 3,050,494)	493.40 (450.23 to 539.26)	4,156,665 (3,725,128 to 4,612,421)	383.47 (346.65 to 423.88)	−0.805 (−0.811 to −0.799)	47,137 (29,475 to 70,001)	8.32 (5.17 to 12.49)	67,143 (42,230 to 101,645)	5.98 (3.73 to 9.09)	−1.052 (−1.059 to −1.046)
Western Sub-Saharan Africa	1,232,370 (1,100,877 to 1,378,607)	1309.22 (1159.08 to 1464.82)	3,002,279 (2,723,834 to 3,278,186)	1340.06 (1211.16 to 1473.27)	0.076 (0.071 to 0.08)	45,885 (31,014 to 65,723)	48.75 (32.77 to 70.46)	99,278 (65,592 to 142,655)	44.43 (29.57 to 63.25)	−0.298 (−0.306 to −0.291)

AAPC, average annual percentage change; CI, confidence interval; CKD, chronic kidney disease; UI, uncertainty interval; YLDs, years lived with disability.

### Regional level

In 2021, the highest ASPR per 100,000 population for anemia attributed to CKD was observed in Central Asia [1615.07 (95% UI: 1455.29 to 1800.63)], followed by Central Sub-Saharan Africa [1531.84 (95% UI: 1329.91 to 1743.46)] and Western Sub-Saharan Africa [1340.06 (95% UI: 1211.16 to 1473.27)]. Western Europe [383.47 (95% UI: 346.65 to 423.88)], Southern Latin America [402.33 (95% UI: 353.04 to 463.19)], and Andean Latin America [409.48 (95% UI: 362.36 to 461.34)] had the lowest ASPR per 100,000 population. The majority of GBD regions showed downward trends in the ASPR from 1990 to 2021, with the most pronounced decrease being found in East Asia [AAPC = −1.626 (95% CI: −1.653 to −1.603)], Andean Latin America [AAPC = −1.061 (95% CI: −1.073 to −1.051)], and High-income Asia Pacific [AAPC = −0.984 (95% CI: −1.002 to −0.97)]. During the same period, all GBD regions showed downward trends in the age-standardized YLD rate, except High-income North America [AAPC = 0.339 (95% CI: 0.318 to 0.356)]. The largest decrease in the age-standardized YLD rate was found in the same regions with the most pronounced decrease in ASPR, while the highest and lowest age-standardized YLD rates were also found in the same regions with the highest and lowest ASPR (Table 1).

### National level

**Figure 2:**
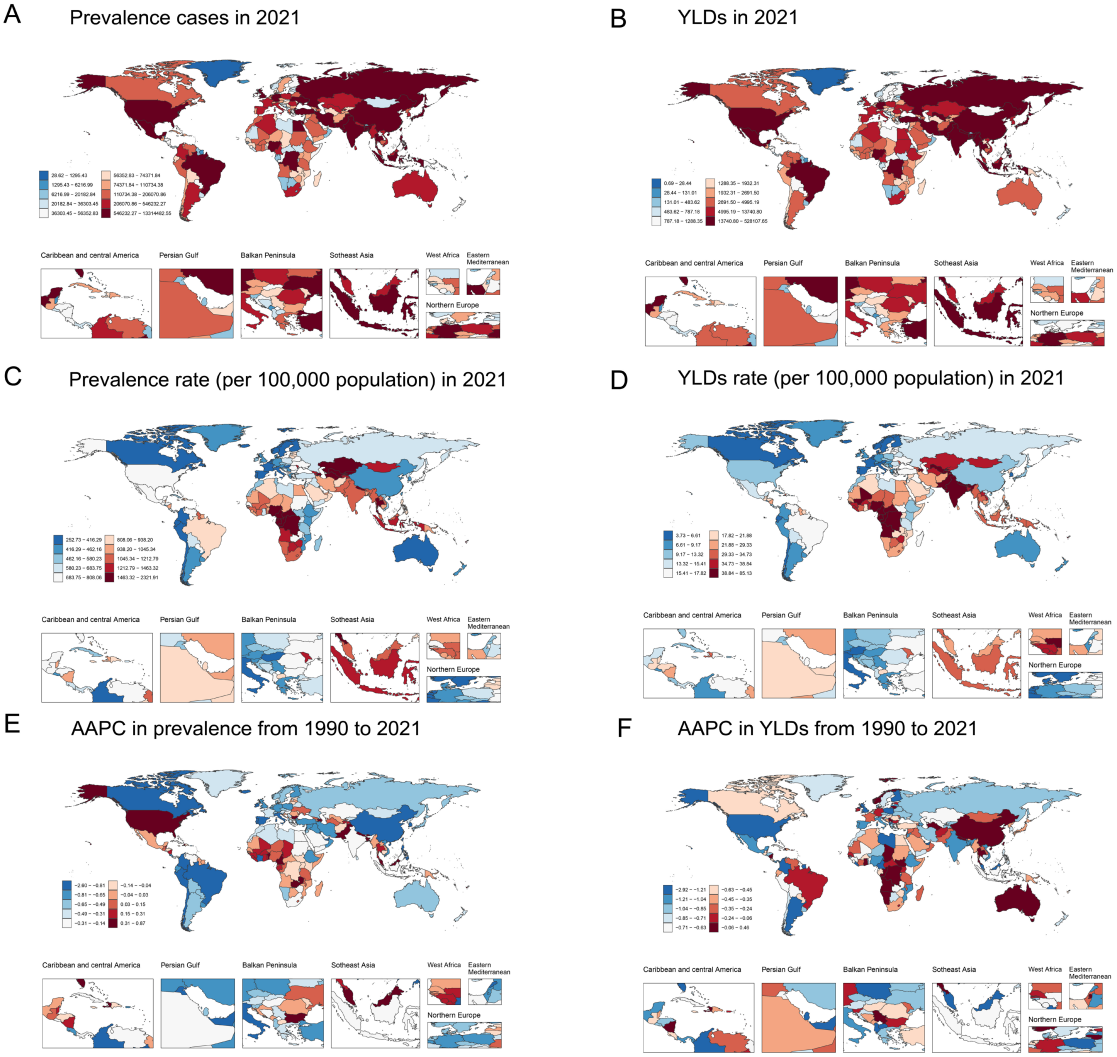
Global map of 2021 anemia attributed to CKD. **(A)** Prevalence cases, **(B)** YLDs, **(C)** age-standardized prevalence rate per 100,000, and **(D)** age-standardized YLD rate per 100,000; AAPC in **(E)** age-standardized prevalence rate and **(F)** age-standardized YLD rate from 1990 to 2021. AAPC, average annual percentage change; CKD, chronic kidney disease; YLDs, years lived with disability.

Details of the burden of anemia attributed to CKD in various countries and territories are presented in Supplementary Table S1 and Figure 2. In 2021, the national ASPR per 100,000 population of anemia attributed to CKD ranged from 255.29 to 1832.39. Nepal [2298.92 (95% UI: 1904.13 to 2765.01)], Uzbekistan [1834.86 (95% UI: 1542.38 to 2205.31)], and Azerbaijan [1759.88 (95% UI: 1469.43 to 2127.35)] had the highest ASPR per 100,000 population in 2021. In contrast, Iceland [255.29 (95% UI: 210.36 to 314.62)], Canada [274.56 (95% UI: 224.78 to 340.89)], and France [290.97 (95% UI: 238.01 to 362.81)] had the lowest ASPR per 100,000 population. The ASPR of anemia attributed to CKD increased in approximately half of all national regions from 1990 to 2021. The most pronounced increase in ASPR was observed in Fiji [AAPC = 0.864 (95% CI: 0.847 to 0.879)], Benin [AAPC = 0.735 (95% CI: 0.729 to 0.740)], and Nepal [AAPC = 0.732 (95% CI: 0.719 to 0.746)]. Conversely, ASPR decreased in more than half of all national regions from 1990 to 2021, with Korea [AAPC = −2.569 (95% CI: −2.597 to −2.552)], China [AAPC = −1.668 (95% CI: 1.703 to −1.644)], and Singapore [AAPC = −1.516 (95% CI: −1.525 to −1.505)] having the most pronounced decrease. Nationally, the age-standardized YLD rate per 100,000 population of anemia attributed to CKD varied from 3.77 to 84.28 in 2021. The highest and lowest age-standardized rates were found in the same regions with the highest and lowest ASPR, while the largest decrease in age-standardized YLD rate was also found in the same regions with the most pronounced decrease in ASPR.

### Age and sex patterns

**Figure 3:**
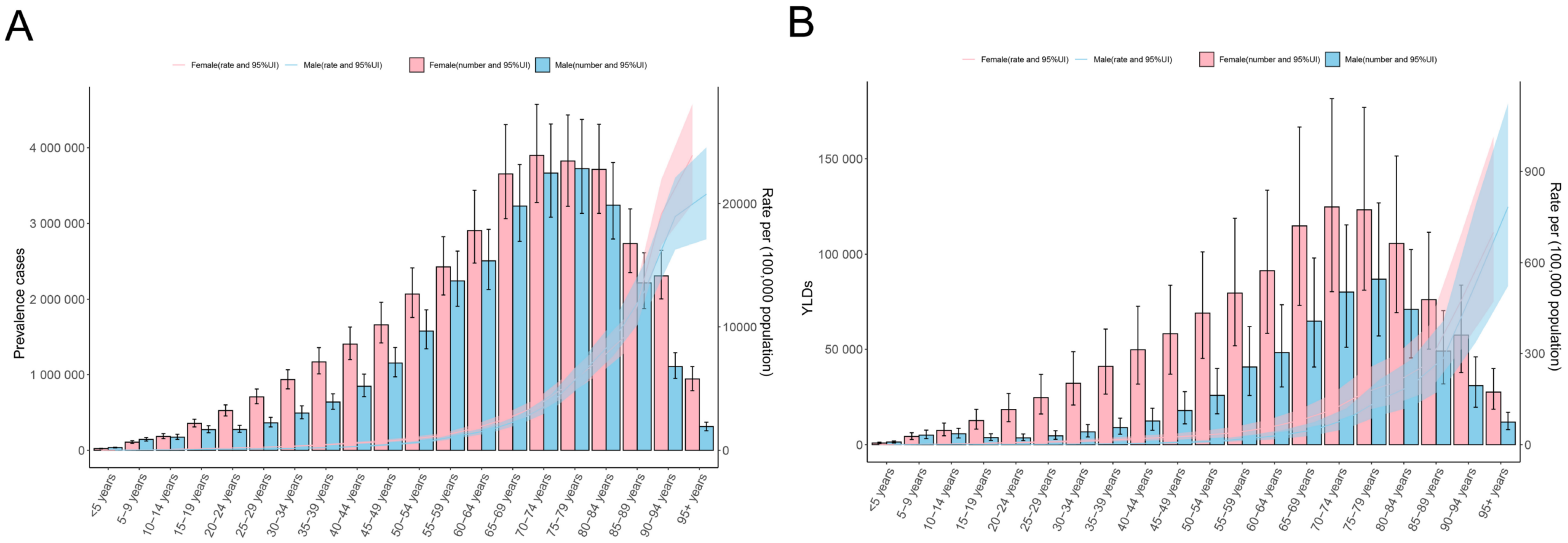
Age-specific counts and the rate of anemia attributed to CKD by sex, 2021. **(A)** Prevalence and **(B)** YLD rates. APC, annual percentage change; CKD, chronic kidney disease; YLDs, years lived with disability.

In 2021, the ASPR and age-standardized YLD rates were 748.10 (95% UI: 691.69 to 807.21) and 15.81 (95% UI: 10.36 to 22.71) for men and 784.53 (95% UI: 728.64 to 842.42) and 24.83 (95% UI: 16.56 to 35.73) for women, respectively. The ASPR and age-standardized YLD rates of women were higher than those of men among most age groups. The number of prevalence cases and YLDs showed a unimodal distribution in men and women, and both peaked in the 70–79 years’ group. For both men and women, ASPR and age-standardized YLD rates increased with age (Table 1; Figures 3A,B).

### Association with the socio-demographic index

**Figure 4:**
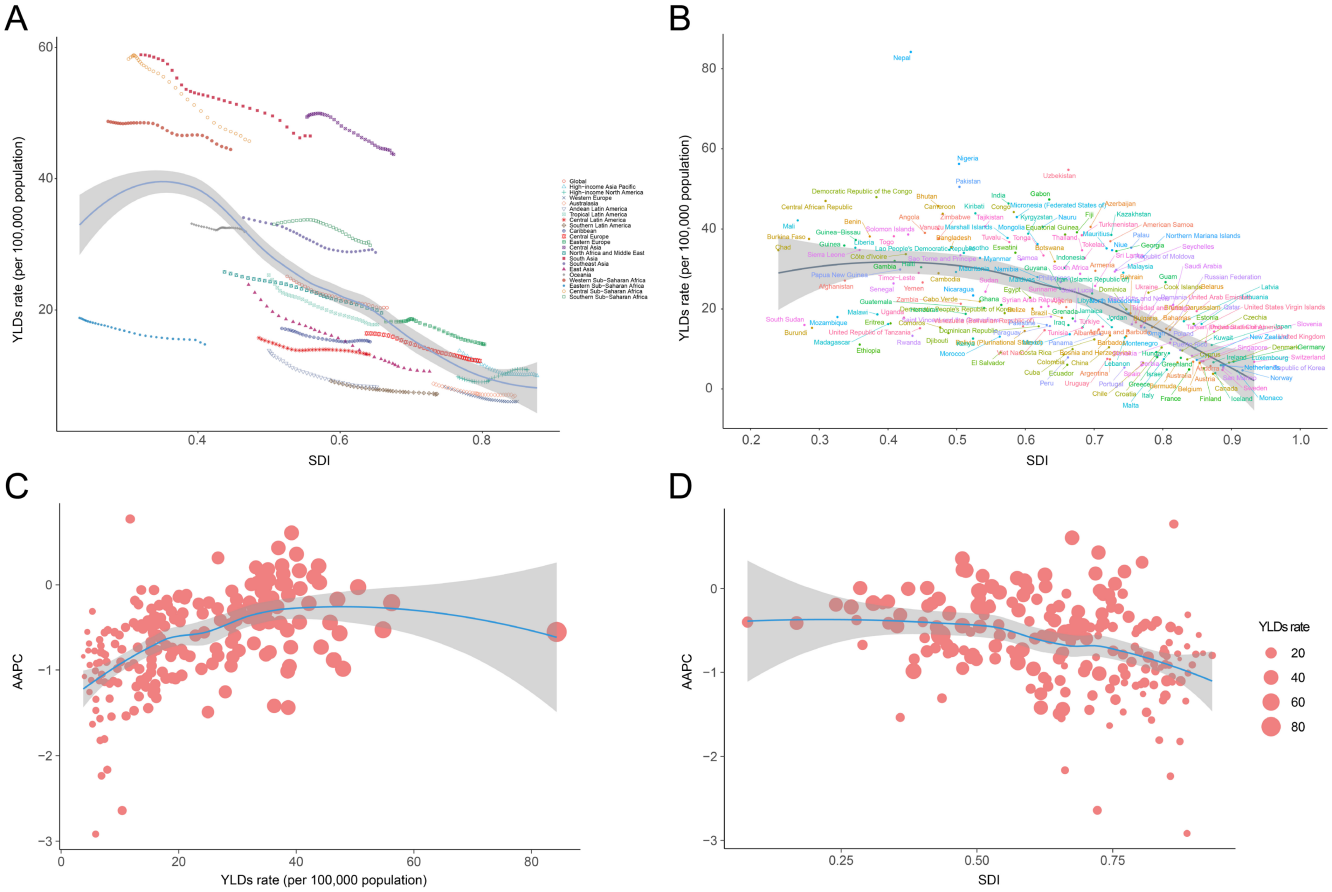
Correlation between SDI and anemia burden attributed to CKD by 21 GBD regions and 204 nations. **(A)** Trends in age-standardized YLD rate across 21 GBD regions, correlated with SDI from 1990 to 2021, **(B)** age-standardized YLD rate at the national level in relation to SDI in 2021, **(C)** AAPC of age-standardized YLD rate at the national level in relation to YLD rate of 2021, and **(D)** AAPC of age-standardized YLD rate at the national level in relation to SDI of 2021. GBD, Global Burden of Disease Study; SDI, socio-demographic index; CKD, chronic kidney disease; YLDs, years lived with disability.

Across all five SDI regions, the low SDI regions had the highest age-standardized YLD rate per 100,000 population [38.72 (95% UI: 26.28 to 54.37)], and the age-standardized YLD rate decreased as SDI increased in 2021 (Table 1). At the regional level, a negative correlation was observed between SDI and the age-standardized YLD rate of anemia attributed to CKD from 1990 to 2019 (Table 1; Figure 4A). The association between SDI and anemia burden attributed to CKD at the national level was consistent with that at the regional level: countries with higher SDI tended to have lower age-standardized YLD rates (Supplementary Table S1; Figure 4B). In addition, at the national level, the AAPC of age-standardized YLD rate was positively correlated with the 2021 age-standardized YLD rate and negatively correlated with the 2021 SDI (Figures 4C,D).

### Predictions of anemia burden attributed to CKD

**Figure 5:**
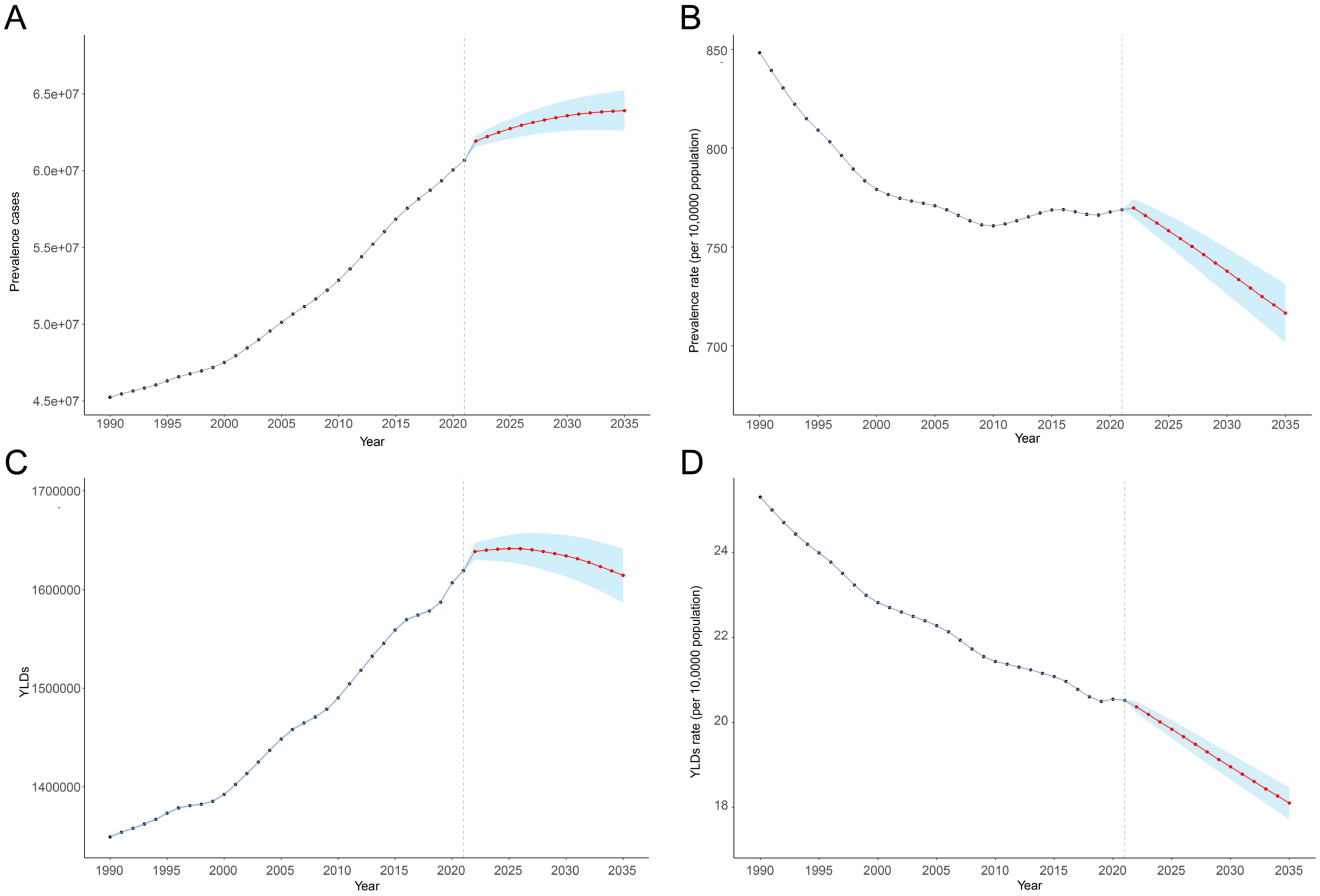
Anemia attributed to CKD from 1990 to 2021 and its predictions for 2035. **(A)** Prevalence cases, **(B)** age-standardized prevalence rate per 100,000, **(C)** YLD rates, and **(D)** age-standardized YLD rate per 100,000. The known data from 1990 to 2021 and predicted data from 2022 to 2030 were divided by a gray dashed line. The shaded area represents 95% uncertainty intervals. APC, annual percentage change; CKD, chronic kidney disease; YLDs, years lived with disability.

Using BAPC analysis, we estimated that the prevalence of anemia cases attributed to CKD will consistently increase from 2022 to 2035, while YLDs are projected to rise initially and then gradually decline. Globally, there will be 63.92 million (95% UI: 60.11 to 67.74) prevalence cases and 1.61 million (95% UI: 1.54 to 1.69) YLDs in 2035. During the same period, the ASPR and age-standardized YLD rates are projected to decrease consistently. Globally, the ASPR and age-standardized YLD rates per 100,000 population are estimated to be 716.52 (95% UI: 673.75 to 759.30) and 18.10 (95% UI: 17.22 to 18.98) in 2035, representing declines of 5.72 and 11.01%, respectively, compared with 2021 (Supplementary Table S2; Figure 5).

### Decomposition analysis of anemia attributed to CKD from 1990 to 2021

**Figure 6:**
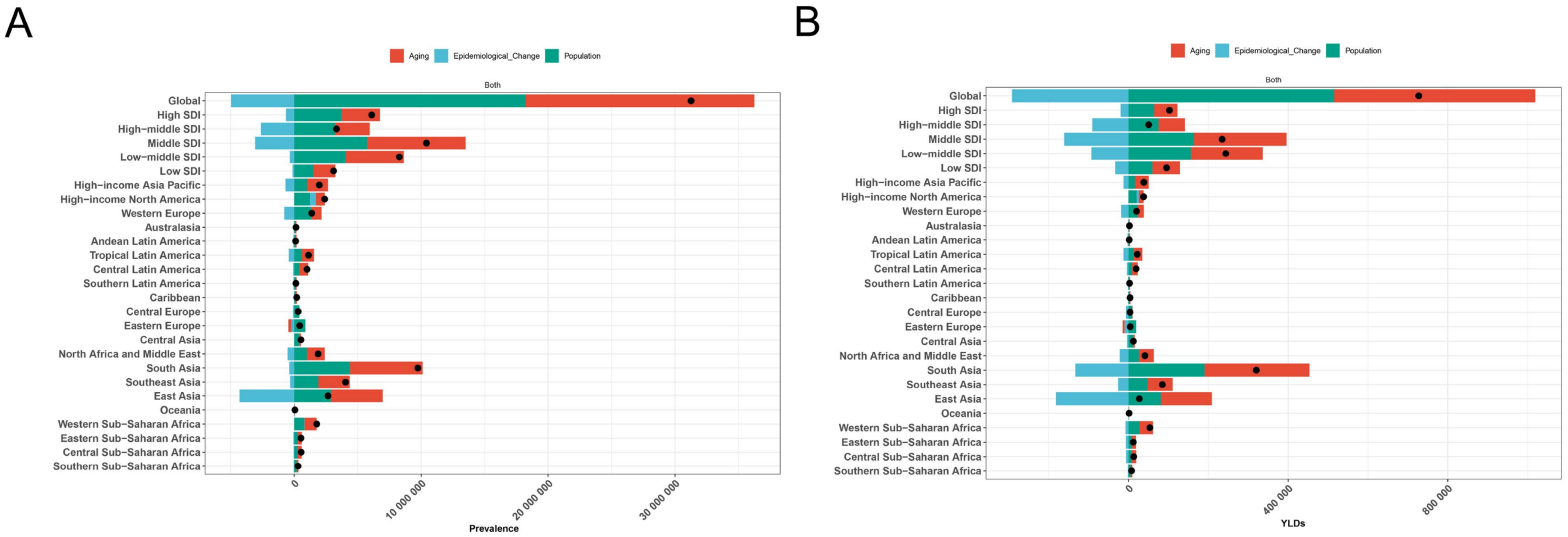
Decomposition analysis of anemia attributed to CKD. **(A)** Prevalence and **(B)** YLD rate. APC, annual percentage change; CKD, chronic kidney disease; YLDs, years lived with disability.

Our decomposition analysis provided insights into the relative contributions of aging, population, and demographically adjusted changes in epidemiology to the prevalence cases and YLDs of anemia attributed to CKD. At the global level and at the five SDI regional levels, although demographically adjusted epidemiological changes decreased the anemia burden attributed to CKD, aging and population growth ultimately led to increases in prevalence and YLDs between 1990 and 2019. Similarly, all 21 GBD regions experienced rises in prevalence and YLDs during this period, primarily driven by aging and population growth, with South Asia showing the highest increases in prevalence and YLDs (Figure 6).

## Discussion

CKD is a major global health issue affecting millions of people and placing a significant strain on healthcare systems. Anemia, a common complication of CKD, further increases the mortality associated with this condition (5–8). However, research specifically focused on the anemia burden attributed to CKD remains limited. To our knowledge, this is the first study to provide a comprehensive analysis of the global, regional, and national anemia burden attributed to CKD from 1990 to 2021 using data from the GBD 2021 study.

Globally, the prevalent cases of anemia attributed to CKD increased by 96.24% from 1990 to 2021, reaching 63.75 million in 2021. This condition accounted for approximately 1.70 million YLDs in 2021, representing a 74.78% increase since 1990. Decomposition analysis indicated that this increase in absolute numbers of prevalence and YLDs was largely driven by population growth and aging, both of which are known contributors to the increasing burden of CKD (35). Consequently, the rising prevalence of CKD^36^ has led to a corresponding increase in anemia attributed to CKD. Notably, although the ASPR for CKD showed a slight decrease, and the age-standardized disability-adjusted life years rate for CKD increased between 1990 and 2021 (36), the ASPR and age-standardized YLD rates for anemia attributed to CKD declined markedly during the same period. This suggests that, despite the growing burden of CKD, efforts in screening, prevention, and treatment of anemia attributed to CKD have helped reduce its specific burden (37–39). Additionally, although the global ASPR of anemia attributed to CKD decreased from 1990 to 2021, this decline stalled after 2010, with a slight increase observed between 2010 and 2015—primarily due to rising ASPR in high-SDI regions. This trend aligns with changes in the ASPR of CKD in high-SDI regions (36), where the increasing ASPR is largely attributed to diabetes and hypertension (40, 41), the most well-established drivers of CKD (42).

At the regional and national levels, the anemia burden attributed to CKD varied substantially. Our analysis revealed a clear negative correlation between SDI and the anemia burden attributed to CKD, with low-SDI regions experiencing higher age-standardized YLD rates. This disparity is largely due to lower per capita income, limited access to healthcare, and poorer educational outcomes in low- and middle-income countries (23). Moreover, at the national level, a negative correlation was observed between the AAPC of age-standardized YLD rates and the SDI in 2021, with high-SDI regions showing more pronounced declines. This likely reflects advancements in treatment and improvements in healthcare quality related to anemia attributed to CKD in high-income countries. These correlations highlight inequities in healthcare resources and variations in the effectiveness of public health interventions across regions, underscoring the need to address socioeconomic disparities as part of comprehensive strategies to prevent and control anemia attributed to CKD.

The anemia burden attributed to CKD also differed by gender and age. In 2021, both the ASPR and age-standardized YLD rates for anemia attributed to CKD increased with age in both men and women. This pattern aligns with the natural decline in kidney function associated with aging, influenced by factors such as nephron count at birth, genetic predisposition, and environmental exposures (35). Women exhibited higher ASPR and age-standardized YLD rates than men across most age groups. This gender disparity may be due to differences in baseline kidney function, hormonal influences, and access to healthcare (43). These findings suggest that greater attention should be directed toward women and older populations.

The results of this study have several important implications for public health interventions aimed at reducing the anemia burden attributed to CKD. First, early detection and management of CKD are essential for reducing the incidence of anemia. This includes regular kidney function screening, especially in high-risk groups such as individuals with diabetes and hypertension (42). Second, in low-SDI regions, where the anemia burden attributed to CKD is highest, efforts should focus on strengthening healthcare infrastructure, improving access to essential medications, and enhancing the capacity of healthcare providers to manage CKD and its complications. Furthermore, the stagnation in the decline in prevalence since 2010 underscores the need for more effective and sustainable interventions.

While this study provides valuable insights into the global anemia burden attributed to CKD, it still has several limitations. First, the accuracy of our estimates depends on the quality and availability of data, which varies across countries and regions. For example, data from low-SDI regions may be less reliable due to limited healthcare infrastructure and incomplete reporting systems. Second, the GBD study relies on modeling techniques to estimate anemia prevalence and YLDs, which may introduce uncertainty. Third, attributing each case of anemia to a single underlying cause is a limitation, as many individuals with anemia likely have multiple comorbid conditions contributing to their diagnosis. Finally, although this study assessed trends and projected future burden based on GBD 2021, the inherent time lag in the database (currently including data only up to 2021) may affect the accuracy of predictions.

In conclusion, despite a global decline in ASPR and age-standardized YLD rates over the past three decades, the anemia burden attributed to CKD remains a significant public health challenge, particularly in low- and middle-income countries. Additionally, women and older adults bear a higher anemia burden attributed to CKD. These findings provide valuable insights for targeting intervention and prevention efforts.

## Data Availability

The original contributions presented in the study are included in the article/Supplementary material, further inquiries can be directed to the corresponding authors.

## Footnotes

^1^ https://vizhub.healthdata.org/gbd-results/
