# Supplementary material for: Global burden of anemia attributed to chronic kidney disease: prevalence, years lived with disability, and predictions to 2035 (Global burden of disease 2021)
Source: Front Nutr. 2025 Nov 18;12:1690686. doi: 10.3389/fnut.2025.1690686 (PMC12669016; doi:10.3389/fnut.2025.1690686)
Supplement: Supplementary file 1 [file Table_1.docx]

Table S1 The prevalence and YLDs of anemia of CKD, and their temporal trends from 1990 to 2021 at the national levels.

| Location | 1990 | | 2021 | | 1990-2021 | 1990 | | 2021 | | 1990-2021 |
| --- | --- | --- | --- | --- | --- | --- | --- | --- | --- | --- |
|  | Prevalence cases (95% UI) | Prevalence rate per 100,000 (95% UI) | Prevalence cases (95% UI) | Prevalence rate per 100,000 (95% UI) | AAPC (%), (95% CI) | YLDs (95% UI) | YLDs rate per 100,000 (95% UI) | YLDs (95% UI) | YLDs rate per 100,000 (95% UI) | AAPC (%), (95% CI) |
| Afghanistan | 58926 (48916 to 72184) | 890.69 (747.01 to 1091.71) | 89992 (75923 to 107028) | 855.95 (694.88 to 1077.84) | -0.126 (-0.132 to -0.12) | 1971 (1279 to 2878) | 30.59 (20.20 to 44.34) | 2730 (1775 to 3972) | 27.08 (17.58 to 38.88) | -0.39 (-0.397 to -0.384) |
| Albania | 20542 (17385 to 24362) | 882.46 (752.13 to 1051.74) | 29339 (24681 to 35364) | 755.96 (651.20 to 885.97) | -0.496 (-0.507 to -0.485) | 487 (304 to 715) | 21.87 (13.68 to 32.19) | 574 (356 to 875) | 14.81 (9.28 to 22.75) | -1.261 (-1.275 to -1.247) |
| Algeria | 128831 (106232 to 152371) | 941.32 (776.51 to 1115.92) | 289978 (240037 to 347889) | 829.90 (684.25 to 990.78) | -0.401 (-0.409 to -0.394) | 3482 (2299 to 5105) | 26.01 (16.89 to 37.95) | 7137 (4639 to 10628) | 20.47 (13.45 to 30.46) | -0.765 (-0.776 to -0.753) |
| American Samoa | 296 (249 to 354) | 1283.51 (1065.25 to 1527.43) | 674 (556 to 809) | 1463.32 (1224.33 to 1751.16) | 0.423 (0.417 to 0.429) | 7 (4 to 10) | 33.43 (21.81 to 50.05) | 16 (10 to 24) | 36.96 (23.41 to 55.92) | 0.325 (0.317 to 0.333) |
| Andorra | 221 (179 to 272) | 461.96 (373.00 to 564.36) | 652 (512 to 802) | 374.38 (302.50 to 460.66) | -0.675 (-0.685 to -0.667) | 3 (2 to 5) | 7.32 (4.45 to 11.43) | 10 (6 to 16) | 5.70 (3.37 to 9.26) | -0.805 (-0.816 to -0.793) |
| Angola | 61793 (51839 to 72466) | 1439.83 (1228.14 to 1713.47) | 192281 (159401 to 229551) | 1446.57 (1194.90 to 1731.82) | 0.015 (0 to 0.028) | 2000 (1276 to 2896) | 48.38 (31.50 to 68.77) | 4995 (3207 to 7365) | 39.05 (24.98 to 56.89) | -0.688 (-0.7 to -0.679) |
| Antigua and Barbuda | 489 (395 to 595) | 877.07 (708.48 to 1075.85) | 847 (697 to 1050) | 843.35 (701.31 to 1031.43) | -0.133 (-0.143 to -0.124) | 9 (6 to 14) | 16.63 (10.64 to 25.39) | 14 (8 to 22) | 14.54 (8.78 to 22.21) | -0.437 (-0.448 to -0.428) |
| Argentina | 154599 (131073 to 184276) | 499.14 (426.35 to 594.31) | 234796 (195067 to 281470) | 419.64 (350.49 to 504.61) | -0.571 (-0.586 to -0.558) | 2659 (1683 to 4084) | 9.09 (5.81 to 13.89) | 4238 (2649 to 6683) | 7.38 (4.62 to 11.64) | -0.688 (-0.706 to -0.671) |
| Armenia | 34381 (28598 to 41141) | 1312.68 (1092.07 to 1551.49) | 54574 (45958 to 66438) | 1301.93 (1103.18 to 1566.28) | -0.026 (-0.03 to -0.022) | 904 (580 to 1354) | 34.25 (22.12 to 50.94) | 1228 (758 to 1835) | 29.51 (18.47 to 44.34) | -0.48 (-0.489 to -0.471) |
| Australia | 91680 (75686 to 114550) | 496.25 (410.65 to 617.28) | 212176 (166957 to 269463) | 413.78 (331.21 to 523.91) | -0.612 (-0.683 to -0.568) | 1570 (987 to 2347) | 8.65 (5.48 to 12.73) | 3515 (2120 to 5478) | 6.77 (4.12 to 10.43) | -0.811 (-0.879 to -0.771) |
| Austria | 55384 (45462 to 68601) | 476.98 (398.86 to 578.52) | 88909 (71993 to 113007) | 417.41 (342.71 to 520.86) | -0.421 (-0.437 to -0.407) | 959 (576 to 1479) | 8.22 (4.92 to 12.64) | 1428 (856 to 2231) | 6.55 (3.96 to 10.25) | -0.728 (-0.742 to -0.712) |
| Azerbaijan | 79643 (67643 to 95788) | 1595.43 (1354.94 to 1954.43) | 171384 (142066 to 211457) | 1759.88 (1469.43 to 2127.35) | 0.321 (0.31 to 0.33) | 2335 (1492 to 3497) | 45.13 (28.99 to 66.32) | 4099 (2606 to 6134) | 40.54 (25.76 to 60.93) | -0.35 (-0.363 to -0.338) |
| Bahamas | 1431 (1179 to 1722) | 879.65 (714.55 to 1063.70) | 3243 (2662 to 3911) | 830.76 (688.29 to 999.37) | -0.183 (-0.194 to -0.173) | 27 (17 to 43) | 16.95 (10.57 to 26.91) | 57 (36 to 88) | 14.98 (9.42 to 23.07) | -0.409 (-0.426 to -0.391) |
| Bahrain | 2157 (1830 to 2542) | 997.72 (814.17 to 1201.19) | 7368 (6037 to 8998) | 851.19 (683.97 to 1046.32) | -0.508 (-0.516 to -0.502) | 54 (34 to 80) | 25.90 (16.46 to 38.41) | 158 (99 to 238) | 18.91 (11.75 to 28.92) | -1 (-1.006 to -0.993) |
| Bangladesh | 443232 (381227 to 505768) | 840.75 (716.25 to 964.04) | 1287122 (1112099 to 1529999) | 967.98 (832.79 to 1144.93) | 0.455 (0.444 to 0.465) | 21896 (14977 to 31110) | 44.21 (30.27 to 61.28) | 46056 (30587 to 66970) | 35.48 (23.77 to 51.75) | -0.71 (-0.722 to -0.7) |
| Barbados | 2173 (1801 to 2607) | 738.37 (623.65 to 872.15) | 3529 (2888 to 4378) | 722.77 (599.37 to 876.53) | -0.068 (-0.07 to -0.067) | 40 (24 to 62) | 14.41 (8.93 to 21.98) | 62 (39 to 96) | 13.16 (8.31 to 19.90) | -0.287 (-0.294 to -0.28) |
| Belarus | 104077 (87029 to 129560) | 843.20 (705.90 to 1035.89) | 119957 (97444 to 147842) | 766.32 (631.49 to 930.96) | -0.308 (-0.321 to -0.296) | 2342 (1459 to 3503) | 19.17 (12.02 to 28.49) | 2387 (1466 to 3650) | 15.39 (9.53 to 23.48) | -0.699 (-0.709 to -0.687) |
| Belgium | 73164 (60486 to 90033) | 485.04 (405.75 to 587.54) | 108736 (87449 to 135239) | 386.12 (318.29 to 467.51) | -0.73 (-0.742 to -0.717) | 1337 (806 to 1986) | 8.88 (5.43 to 13.26) | 1855 (1115 to 2846) | 6.47 (3.91 to 9.77) | -1.005 (-1.022 to -0.991) |
| Belize | 976 (831 to 1162) | 942.45 (785.58 to 1143.23) | 3116 (2489 to 3730) | 999.15 (804.11 to 1207.42) | 0.185 (0.179 to 0.19) | 22 (14 to 32) | 20.75 (13.35 to 30.32) | 63 (39 to 94) | 20.07 (12.26 to 29.87) | -0.097 (-0.11 to -0.086) |
| Benin | 17317 (15373 to 19652) | 786.80 (688.44 to 910.31) | 61491 (53526 to 71657) | 987.17 (853.35 to 1148.11) | 0.735 (0.729 to 0.74) | 840 (573 to 1156) | 38.29 (26.12 to 52.84) | 2312 (1519 to 3291) | 38.16 (24.80 to 53.74) | -0.009 (-0.017 to -0.001) |
| Bermuda | 377 (308 to 455) | 637.98 (521.02 to 763.37) | 612 (460 to 769) | 451.55 (351.57 to 557.41) | -1.108 (-1.116 to -1.099) | 6 (4 to 10) | 10.60 (6.46 to 16.49) | 9 (5 to 14) | 6.61 (3.88 to 10.95) | -1.506 (-1.515 to -1.498) |
| Bhutan | 2529 (2174 to 2900) | 946.15 (811.96 to 1096.83) | 6875 (5881 to 8054) | 1091.32 (925.62 to 1283.58) | 0.455 (0.45 to 0.46) | 137 (93 to 193) | 52.06 (35.95 to 72.12) | 278 (183 to 392) | 45.74 (30.17 to 64.17) | -0.418 (-0.423 to -0.414) |
| Bolivia (Plurinational State of) | 28572 (24279 to 32985) | 804.62 (677.81 to 935.23) | 60420 (49519 to 73667) | 683.75 (559.95 to 828.23) | -0.522 (-0.525 to -0.518) | 698 (454 to 1022) | 20.35 (13.25 to 30.08) | 1227 (754 to 1885) | 14.41 (8.90 to 21.84) | -1.111 (-1.116 to -1.106) |
| Bosnia and Herzegovina | 26531 (21748 to 32655) | 667.35 (561.10 to 793.18) | 35396 (29498 to 42588) | 636.97 (535.22 to 754.46) | -0.148 (-0.159 to -0.136) | 639 (404 to 947) | 16.87 (11.01 to 24.72) | 750 (469 to 1112) | 13.32 (8.35 to 19.76) | -0.76 (-0.777 to -0.744) |
| Botswana | 7370 (6194 to 8822) | 1278.50 (1077.61 to 1530.54) | 19199 (15980 to 22916) | 1277.90 (1071.88 to 1526.36) | 0.011 (-0.006 to 0.031) | 206 (133 to 302) | 35.84 (23.21 to 52.47) | 479 (300 to 711) | 31.86 (20.31 to 47.05) | -0.365 (-0.392 to -0.342) |
| Brazil | 949134 (801929 to 1109541) | 1089.82 (920.72 to 1273.01) | 2054503 (1732410 to 2452075) | 837.13 (707.77 to 994.98) | -0.839 (-0.85 to -0.828) | 22272 (14301 to 32853) | 25.45 (16.37 to 37.96) | 43683 (27178 to 67561) | 17.82 (11.08 to 27.60) | -1.141 (-1.153 to -1.129) |
| Brunei Darussalam | 868 (772 to 990) | 793.13 (703.48 to 908.73) | 2341 (1960 to 2851) | 762.11 (637.80 to 939.00) | -0.126 (-0.135 to -0.118) | 16 (10 to 24) | 17.09 (10.73 to 25.00) | 40 (25 to 63) | 14.73 (9.22 to 22.05) | -0.477 (-0.487 to -0.469) |
| Bulgaria | 66527 (54317 to 81541) | 669.29 (557.95 to 799.41) | 88686 (73344 to 107096) | 747.94 (623.01 to 902.52) | 0.362 (0.349 to 0.373) | 1568 (971 to 2383) | 16.15 (10.08 to 23.58) | 1934 (1230 to 2904) | 15.79 (9.88 to 23.41) | -0.07 (-0.083 to -0.053) |
| Burkina Faso | 45347 (38258 to 53450) | 1018.66 (857.44 to 1198.48) | 114723 (96847 to 133008) | 1120.48 (938.95 to 1320.87) | 0.307 (0.3 to 0.312) | 1658 (1078 to 2429) | 37.57 (24.63 to 54.20) | 3803 (2520 to 5672) | 37.53 (25.34 to 55.42) | -0.003 (-0.012 to 0.004) |
| Burundi | 15489 (13298 to 18574) | 598.22 (508.97 to 728.35) | 29385 (24782 to 34458) | 529.40 (442.33 to 637.59) | -0.394 (-0.405 to -0.384) | 444 (284 to 671) | 18.47 (12.04 to 26.97) | 788 (510 to 1174) | 15.22 (10.08 to 22.54) | -0.619 (-0.633 to -0.606) |
| Cabo Verde | 2146 (1800 to 2563) | 907.24 (758.78 to 1088.45) | 4142 (3443 to 4898) | 887.28 (732.80 to 1050.66) | -0.07 (-0.076 to -0.062) | 64 (41 to 95) | 27.22 (17.58 to 40.72) | 102 (65 to 150) | 21.88 (13.97 to 32.20) | -0.695 (-0.706 to -0.682) |
| Cambodia | 46709 (39183 to 56274) | 1011.54 (839.14 to 1226.87) | 114044 (94682 to 138587) | 988.20 (817.54 to 1194.20) | -0.077 (-0.084 to -0.071) | 1683 (1087 to 2379) | 37.57 (24.49 to 53.57) | 3188 (2015 to 4737) | 28.91 (18.42 to 43.06) | -0.842 (-0.85 to -0.834) |
| Cameroon | 57590 (49426 to 66806) | 1237.26 (1059.06 to 1455.74) | 215847 (183596 to 251129) | 1523.65 (1287.94 to 1789.21) | 0.673 (0.657 to 0.689) | 1915 (1262 to 2736) | 41.69 (27.86 to 59.20) | 5951 (3937 to 8901) | 43.80 (28.90 to 64.97) | 0.159 (0.129 to 0.185) |
| Canada | 120905 (98397 to 148218) | 377.26 (309.53 to 460.17) | 189903 (153982 to 240884) | 274.56 (224.78 to 340.89) | -1.016 (-1.039 to -0.996) | 1742 (1051 to 2723) | 5.41 (3.28 to 8.46) | 2773 (1628 to 4436) | 3.77 (2.21 to 6.12) | -1.159 (-1.187 to -1.135) |
| Central African Republic | 18059 (14781 to 21495) | 1509.33 (1241.49 to 1814.71) | 36303 (30698 to 42915) | 1497.14 (1261.30 to 1761.41) | -0.029 (-0.039 to -0.02) | 564 (372 to 820) | 49.30 (32.37 to 70.35) | 1084 (702 to 1597) | 46.99 (30.71 to 69.42) | -0.153 (-0.163 to -0.146) |
| Chad | 27984 (23856 to 32366) | 949.01 (807.17 to 1109.73) | 68433 (58237 to 80032) | 1013.95 (853.72 to 1192.63) | 0.214 (0.211 to 0.218) | 1068 (706 to 1521) | 36.31 (24.12 to 51.52) | 2342 (1539 to 3512) | 34.72 (23.05 to 51.36) | -0.137 (-0.142 to -0.132) |
| Chile | 44908 (37970 to 54488) | 457.68 (386.86 to 556.43) | 91330 (74031 to 114096) | 355.07 (289.00 to 442.90) | -0.811 (-0.832 to -0.788) | 822 (511 to 1228) | 9.29 (5.91 to 13.51) | 1689 (1056 to 2594) | 6.49 (4.06 to 9.99) | -1.163 (-1.181 to -1.143) |
| China | 5645085 (5170014 to 6161351) | 713.99 (653.40 to 779.90) | 8057990 (7313368 to 8886242) | 422.68 (386.50 to 464.60) | -1.668 (-1.703 to -1.644) | 169970 (112856 to 240346) | 23.89 (15.99 to 34.01) | 191114 (124978 to 274103) | 10.39 (6.79 to 14.81) | -2.642 (-2.669 to -2.619) |
| Colombia | 111026 (95362 to 129844) | 597.00 (509.15 to 706.46) | 233951 (196391 to 281664) | 416.29 (349.29 to 505.64) | -1.156 (-1.161 to -1.152) | 2479 (1600 to 3661) | 14.27 (9.26 to 21.14) | 4793 (2911 to 7234) | 8.56 (5.16 to 12.96) | -1.635 (-1.646 to -1.626) |
| Comoros | 1456 (1222 to 1693) | 628.22 (533.84 to 728.73) | 2963 (2470 to 3518) | 573.31 (480.02 to 680.85) | -0.297 (-0.304 to -0.29) | 43 (27 to 63) | 19.21 (12.61 to 27.58) | 75 (47 to 114) | 15.34 (9.81 to 22.99) | -0.722 (-0.742 to -0.705) |
| Congo | 16174 (13515 to 19403) | 1453.28 (1221.12 to 1740.79) | 44037 (36630 to 53233) | 1499.86 (1241.39 to 1789.61) | 0.098 (0.087 to 0.106) | 595 (391 to 845) | 54.08 (35.44 to 76.24) | 1288 (833 to 1926) | 44.22 (28.18 to 65.22) | -0.649 (-0.659 to -0.641) |
| Cook Islands | 135 (113 to 163) | 1116.13 (920.44 to 1357.66) | 290 (232 to 359) | 1147.65 (929.32 to 1399.28) | 0.086 (0.073 to 0.096) | 3 (2 to 4) | 27.34 (17.40 to 39.63) | 6 (4 to 9) | 24.07 (15.06 to 35.79) | -0.415 (-0.428 to -0.401) |
| Costa Rica | 16149 (13685 to 18955) | 850.91 (712.59 to 1005.87) | 36559 (30407 to 43780) | 670.09 (556.08 to 812.30) | -0.737 (-0.772 to -0.685) | 307 (193 to 460) | 17.07 (10.74 to 25.31) | 674 (414 to 1032) | 12.34 (7.63 to 18.90) | -1.032 (-1.075 to -0.972) |
| Croatia | 30765 (25742 to 36759) | 561.28 (473.67 to 663.52) | 44437 (36099 to 54254) | 528.03 (438.64 to 642.97) | -0.196 (-0.202 to -0.19) | 676 (437 to 1028) | 12.64 (8.15 to 19.34) | 876 (560 to 1314) | 10.33 (6.57 to 15.38) | -0.648 (-0.658 to -0.636) |
| Cuba | 65305 (53172 to 79678) | 649.65 (531.02 to 789.56) | 110734 (88430 to 135237) | 569.00 (462.48 to 695.95) | -0.424 (-0.43 to -0.419) | 1147 (698 to 1787) | 12.08 (7.44 to 18.86) | 1951 (1120 to 3099) | 9.92 (5.87 to 15.47) | -0.631 (-0.642 to -0.622) |
| Cyprus | 4603 (3723 to 5626) | 658.93 (549.50 to 791.19) | 8809 (7000 to 11024) | 459.57 (374.87 to 559.35) | -1.152 (-1.162 to -1.142) | 76 (46 to 118) | 11.98 (7.56 to 17.94) | 141 (85 to 217) | 7.36 (4.43 to 11.22) | -1.564 (-1.585 to -1.547) |
| Czechia | 72080 (60614 to 86182) | 594.99 (498.75 to 716.22) | 98759 (80209 to 119731) | 500.60 (410.98 to 611.63) | -0.556 (-0.564 to -0.549) | 1611 (1028 to 2443) | 13.25 (8.43 to 19.95) | 1954 (1192 to 3074) | 9.56 (5.87 to 14.98) | -1.037 (-1.048 to -1.028) |
| Côte d'Ivoire | 53062 (46067 to 61732) | 1090.85 (926.36 to 1294.11) | 139557 (117765 to 165030) | 1099.69 (907.19 to 1308.61) | 0.026 (0.017 to 0.033) | 1883 (1265 to 2720) | 38.47 (25.38 to 54.85) | 4268 (2792 to 6192) | 33.76 (22.31 to 48.93) | -0.419 (-0.426 to -0.411) |
| Democratic People's Republic of Korea | 116589 (99345 to 137643) | 739.83 (635.13 to 869.36) | 206009 (174586 to 246560) | 669.51 (574.07 to 789.92) | -0.324 (-0.33 to -0.319) | 3307 (2121 to 4892) | 23.55 (15.26 to 34.45) | 5584 (3635 to 8097) | 18.97 (12.22 to 27.68) | -0.697 (-0.705 to -0.69) |
| Democratic Republic of the Congo | 275350 (226854 to 330271) | 1627.25 (1362.56 to 1925.66) | 627972 (531800 to 736232) | 1564.49 (1305.57 to 1840.03) | -0.127 (-0.136 to -0.115) | 10436 (6909 to 14994) | 61.90 (41.08 to 88.01) | 18713 (12016 to 27358) | 47.95 (31.11 to 70.16) | -0.824 (-0.832 to -0.814) |
| Denmark | 42902 (34845 to 52203) | 512.29 (420.41 to 614.53) | 57400 (46845 to 71719) | 437.37 (359.86 to 539.36) | -0.504 (-0.511 to -0.498) | 705 (429 to 1124) | 8.37 (5.13 to 13.22) | 872 (516 to 1372) | 6.55 (3.87 to 10.38) | -0.786 (-0.794 to -0.779) |
| Djibouti | 1071 (900 to 1277) | 583.61 (491.58 to 689.07) | 4163 (3429 to 5223) | 567.14 (473.14 to 696.56) | -0.091 (-0.1 to -0.083) | 28 (18 to 43) | 16.56 (10.80 to 24.78) | 92 (57 to 138) | 13.91 (8.76 to 20.57) | -0.554 (-0.565 to -0.545) |
| Dominica | 590 (483 to 709) | 989.99 (815.12 to 1181.85) | 817 (660 to 991) | 1020.54 (834.72 to 1223.17) | 0.096 (0.09 to 0.1) | 12 (8 to 18) | 21.25 (13.62 to 31.57) | 16 (9 to 23) | 19.92 (12.20 to 29.69) | -0.213 (-0.22 to -0.207) |
| Dominican Republic | 32346 (26693 to 39351) | 841.69 (692.99 to 1040.24) | 82430 (66106 to 101004) | 819.61 (654.94 to 1001.67) | -0.091 (-0.103 to -0.081) | 716 (458 to 1082) | 19.16 (12.31 to 28.72) | 1567 (991 to 2425) | 15.63 (9.98 to 24.24) | -0.657 (-0.669 to -0.645) |
| Ecuador | 24790 (20824 to 28954) | 434.46 (359.09 to 517.33) | 56489 (46741 to 69596) | 349.67 (288.95 to 431.52) | -0.705 (-0.724 to -0.689) | 457 (284 to 707) | 8.31 (5.10 to 12.90) | 960 (600 to 1529) | 6.02 (3.79 to 9.53) | -1.023 (-1.043 to -0.999) |
| Egypt | 299600 (250935 to 351033) | 1007.56 (833.35 to 1205.01) | 618360 (510166 to 766240) | 959.82 (785.67 to 1173.02) | -0.153 (-0.161 to -0.146) | 8020 (5186 to 11815) | 28.56 (18.68 to 40.92) | 13741 (8857 to 19803) | 22.84 (14.99 to 33.07) | -0.715 (-0.723 to -0.708) |
| El Salvador | 14287 (12341 to 16755) | 455.35 (390.92 to 536.13) | 27796 (23170 to 34048) | 432.19 (358.64 to 531.97) | -0.166 (-0.182 to -0.152) | 367 (237 to 541) | 12.04 (7.80 to 17.96) | 592 (377 to 921) | 9.10 (5.80 to 14.20) | -0.893 (-0.916 to -0.875) |
| Equatorial Guinea | 2877 (2404 to 3414) | 1421.22 (1201.05 to 1691.51) | 9006 (7589 to 10698) | 1490.48 (1242.83 to 1792.25) | 0.152 (0.14 to 0.163) | 111 (73 to 159) | 55.69 (36.95 to 80.23) | 231 (148 to 343) | 38.63 (25.08 to 56.94) | -1.186 (-1.2 to -1.173) |
| Eritrea | 8853 (7490 to 10394) | 618.60 (519.30 to 733.55) | 19128 (16029 to 22704) | 587.01 (489.24 to 706.46) | -0.17 (-0.178 to -0.163) | 275 (174 to 410) | 20.03 (12.75 to 29.62) | 490 (314 to 739) | 16.42 (10.51 to 23.98) | -0.632 (-0.643 to -0.623) |
| Estonia | 17097 (13991 to 21156) | 880.08 (725.48 to 1072.13) | 24354 (19581 to 30654) | 854.41 (697.04 to 1039.21) | -0.091 (-0.101 to -0.082) | 378 (240 to 560) | 19.81 (12.73 to 29.55) | 450 (270 to 697) | 16.31 (10.06 to 24.89) | -0.609 (-0.62 to -0.598) |
| Eswatini | 3769 (3208 to 4463) | 1242.71 (1044.22 to 1468.55) | 6974 (5864 to 8315) | 1242.26 (1042.37 to 1486.22) | 0.025 (-0.005 to 0.06) | 109 (70 to 158) | 35.77 (23.40 to 51.26) | 192 (123 to 280) | 34.10 (21.92 to 49.60) | -0.115 (-0.143 to -0.089) |
| Ethiopia | 117308 (105865 to 130456) | 555.83 (493.45 to 624.66) | 206071 (184185 to 227455) | 451.20 (400.37 to 507.85) | -0.675 (-0.691 to -0.662) | 3702 (2466 to 5386) | 17.53 (11.60 to 25.51) | 5037 (3290 to 7348) | 11.05 (7.15 to 16.01) | -1.476 (-1.489 to -1.467) |
| Fiji | 4421 (3853 to 5078) | 1163.94 (1001.69 to 1345.08) | 11434 (9584 to 13506) | 1519.83 (1276.77 to 1788.05) | 0.864 (0.847 to 0.879) | 114 (73 to 168) | 34.01 (22.00 to 49.28) | 261 (166 to 396) | 39.19 (25.30 to 59.28) | 0.457 (0.442 to 0.47) |
| Finland | 27901 (23186 to 33500) | 406.66 (338.82 to 485.70) | 48805 (38821 to 60517) | 319.35 (262.95 to 387.03) | -0.764 (-0.774 to -0.753) | 492 (317 to 743) | 7.18 (4.62 to 10.82) | 793 (478 to 1268) | 5.13 (3.16 to 8.20) | -1.075 (-1.09 to -1.06) |
| France | 276097 (220781 to 339892) | 322.57 (263.90 to 391.71) | 538033 (426115 to 690106) | 290.97 (238.01 to 362.81) | -0.345 (-0.366 to -0.322) | 5119 (3067 to 7861) | 6.01 (3.57 to 9.25) | 9078 (5359 to 14494) | 4.86 (2.90 to 7.70) | -0.698 (-0.72 to -0.677) |
| Gabon | 8061 (6769 to 9694) | 1401.37 (1172.26 to 1688.58) | 16357 (13738 to 18977) | 1498.11 (1245.19 to 1764.33) | 0.211 (0.203 to 0.22) | 320 (215 to 452) | 55.45 (37.54 to 77.80) | 528 (351 to 763) | 47.36 (31.35 to 68.54) | -0.509 (-0.514 to -0.504) |
| Gambia | 3522 (3005 to 4164) | 901.30 (767.73 to 1059.92) | 10427 (8720 to 12438) | 946.02 (787.07 to 1133.49) | 0.16 (0.154 to 0.166) | 135 (90 to 194) | 33.82 (22.86 to 48.16) | 356 (234 to 523) | 32.05 (20.79 to 46.85) | -0.163 (-0.173 to -0.153) |
| Georgia | 80713 (65986 to 98638) | 1376.88 (1145.27 to 1661.79) | 83473 (67730 to 102582) | 1397.41 (1157.44 to 1701.02) | 0.046 (0.041 to 0.051) | 2131 (1353 to 3179) | 36.07 (23.02 to 53.54) | 2039 (1268 to 3055) | 34.49 (21.58 to 51.59) | -0.146 (-0.154 to -0.141) |
| Germany | 684736 (562228 to 814078) | 560.76 (468.01 to 659.00) | 986751 (805086 to 1236184) | 456.65 (383.62 to 551.04) | -0.652 (-0.667 to -0.639) | 11426 (7101 to 17198) | 9.12 (5.73 to 13.98) | 15538 (9323 to 24271) | 6.80 (4.14 to 10.70) | -0.932 (-0.942 to -0.92) |
| Ghana | 41592 (34400 to 48749) | 602.27 (493.19 to 708.87) | 121764 (100140 to 142715) | 647.15 (536.03 to 773.59) | 0.237 (0.228 to 0.246) | 1562 (1029 to 2250) | 22.84 (15.04 to 32.33) | 3834 (2445 to 5665) | 21.02 (13.38 to 30.65) | -0.257 (-0.269 to -0.243) |
| Greece | 73523 (62093 to 86323) | 526.92 (450.19 to 617.58) | 131412 (105603 to 162107) | 457.83 (383.12 to 553.70) | -0.444 (-0.452 to -0.435) | 1320 (831 to 1966) | 9.54 (6.01 to 14.24) | 2388 (1487 to 3805) | 7.94 (4.91 to 12.39) | -0.588 (-0.6 to -0.578) |
| Greenland | 164 (135 to 198) | 525.67 (438.95 to 641.90) | 271 (217 to 348) | 462.16 (374.18 to 580.19) | -0.43 (-0.446 to -0.415) | 3 (2 to 4) | 9.98 (6.16 to 15.48) | 4 (3 to 7) | 7.74 (4.73 to 11.78) | -0.82 (-0.839 to -0.803) |
| Grenada | 695 (585 to 818) | 914.68 (769.70 to 1077.15) | 1030 (822 to 1275) | 949.12 (758.11 to 1164.09) | 0.115 (0.11 to 0.119) | 16 (10 to 24) | 20.91 (13.34 to 31.22) | 19 (12 to 29) | 17.63 (10.96 to 27.01) | -0.551 (-0.556 to -0.546) |
| Guam | 796 (646 to 972) | 1086.28 (893.14 to 1330.11) | 2559 (2066 to 3096) | 1242.90 (991.76 to 1488.55) | 0.425 (0.405 to 0.443) | 16 (10 to 25) | 24.21 (15.35 to 37.23) | 59 (37 to 90) | 26.69 (16.77 to 40.89) | 0.308 (0.283 to 0.332) |
| Guatemala | 28480 (24732 to 32971) | 747.19 (644.41 to 867.19) | 87127 (74084 to 101599) | 766.73 (648.32 to 904.92) | 0.082 (0.076 to 0.087) | 882 (578 to 1272) | 24.04 (15.74 to 34.73) | 2289 (1463 to 3430) | 20.23 (12.82 to 30.59) | -0.558 (-0.566 to -0.55) |
| Guinea | 33176 (27924 to 38851) | 977.08 (822.69 to 1150.97) | 66615 (56715 to 77262) | 1054.80 (887.20 to 1237.72) | 0.253 (0.246 to 0.259) | 1277 (855 to 1819) | 38.08 (25.57 to 53.75) | 2207 (1448 to 3213) | 35.90 (23.50 to 51.53) | -0.184 (-0.191 to -0.177) |
| Guinea-Bissau | 4646 (3938 to 5453) | 1047.46 (880.31 to 1254.82) | 9421 (7918 to 11235) | 1074.49 (901.84 to 1303.45) | 0.09 (0.084 to 0.096) | 189 (128 to 276) | 42.23 (28.12 to 62.48) | 323 (207 to 477) | 37.27 (23.83 to 54.40) | -0.4 (-0.406 to -0.395) |
| Guyana | 4715 (4051 to 5446) | 1083.84 (923.93 to 1274.38) | 6910 (5689 to 8324) | 1084.64 (895.67 to 1300.02) | 0.001 (-0.005 to 0.006) | 115 (74 to 170) | 28.02 (18.30 to 40.72) | 157 (99 to 243) | 25.13 (16.10 to 38.28) | -0.358 (-0.367 to -0.349) |
| Haiti | 28441 (24183 to 33993) | 896.42 (749.75 to 1068.94) | 74189 (62103 to 88621) | 999.60 (833.42 to 1191.21) | 0.352 (0.347 to 0.357) | 1085 (726 to 1551) | 33.51 (22.61 to 48.51) | 2202 (1438 to 3243) | 30.42 (20.27 to 44.60) | -0.313 (-0.318 to -0.31) |
| Honduras | 16961 (14624 to 19664) | 762.19 (648.53 to 897.12) | 47045 (40111 to 56159) | 740.64 (625.81 to 886.67) | -0.084 (-0.091 to -0.079) | 450 (294 to 656) | 21.58 (14.05 to 31.17) | 1127 (721 to 1619) | 18.77 (12.14 to 26.91) | -0.445 (-0.45 to -0.44) |
| Hungary | 58401 (50123 to 70202) | 452.91 (388.30 to 540.68) | 71424 (61183 to 84064) | 413.26 (350.02 to 493.14) | -0.288 (-0.297 to -0.28) | 1530 (996 to 2252) | 11.67 (7.53 to 17.34) | 1673 (1069 to 2477) | 9.17 (5.87 to 13.60) | -0.769 (-0.779 to -0.76) |
| Iceland | 1026 (830 to 1246) | 335.94 (273.26 to 406.02) | 1753 (1392 to 2186) | 255.29 (210.36 to 314.62) | -0.876 (-0.893 to -0.858) | 17 (10 to 27) | 5.56 (3.40 to 8.73) | 27 (16 to 44) | 3.95 (2.33 to 6.24) | -1.083 (-1.104 to -1.061) |
| India | 5719581 (5239569 to 6244507) | 1205.00 (1107.60 to 1322.76) | 13182656 (12140711 to 14325056) | 1108.91 (1019.64 to 1206.99) | -0.263 (-0.288 to -0.242) | 275432 (187432 to 381724) | 61.31 (41.90 to 84.69) | 522879 (355089 to 728557) | 46.36 (31.64 to 64.59) | -0.891 (-0.907 to -0.875) |
| Indonesia | 1357287 (1211162 to 1516905) | 1354.45 (1196.72 to 1525.36) | 2903824 (2581487 to 3274575) | 1295.55 (1145.08 to 1476.68) | -0.137 (-0.167 to -0.106) | 33795 (21596 to 49070) | 37.41 (23.92 to 54.00) | 62090 (39041 to 93535) | 30.25 (19.36 to 44.62) | -0.646 (-0.672 to -0.617) |
| Iran (Islamic Republic of) | 349009 (296732 to 407935) | 1306.81 (1121.45 to 1519.71) | 770198 (660221 to 905261) | 1034.59 (875.52 to 1231.02) | -0.742 (-0.754 to -0.73) | 8796 (5679 to 12712) | 33.06 (21.27 to 46.93) | 17953 (11538 to 26031) | 23.87 (15.32 to 34.47) | -1.037 (-1.051 to -1.024) |
| Iraq | 79019 (67929 to 91000) | 823.56 (711.28 to 961.49) | 172077 (144313 to 204047) | 670.65 (565.80 to 805.29) | -0.66 (-0.67 to -0.653) | 2177 (1447 to 3219) | 22.58 (14.86 to 32.98) | 4088 (2615 to 6088) | 15.99 (10.22 to 23.97) | -1.115 (-1.124 to -1.106) |
| Ireland | 26206 (20663 to 33351) | 671.77 (535.10 to 829.36) | 38206 (30964 to 46128) | 464.06 (382.84 to 554.17) | -1.176 (-1.191 to -1.162) | 432 (263 to 650) | 11.36 (6.98 to 17.02) | 553 (327 to 859) | 6.65 (3.97 to 10.38) | -1.703 (-1.726 to -1.684) |
| Israel | 31525 (25466 to 38676) | 683.50 (559.24 to 829.93) | 71004 (58227 to 86097) | 537.36 (441.44 to 644.06) | -0.773 (-0.779 to -0.767) | 549 (326 to 842) | 12.39 (7.50 to 19.02) | 1204 (738 to 1886) | 8.89 (5.43 to 14.04) | -1.06 (-1.071 to -1.05) |
| Italy | 377351 (327523 to 432736) | 455.25 (399.01 to 518.71) | 535154 (436944 to 644098) | 311.84 (263.20 to 369.01) | -1.219 (-1.236 to -1.206) | 6272 (3765 to 9547) | 7.50 (4.58 to 11.27) | 8630 (5357 to 13264) | 4.82 (2.98 to 7.41) | -1.413 (-1.429 to -1.399) |
| Jamaica | 16001 (13247 to 19211) | 858.57 (708.64 to 1035.26) | 26666 (21949 to 32297) | 832.73 (679.84 to 1011.18) | -0.101 (-0.108 to -0.094) | 316 (195 to 486) | 16.94 (10.52 to 26.23) | 527 (326 to 788) | 15.38 (9.31 to 23.12) | -0.314 (-0.321 to -0.307) |
| Japan | 1431258 (1223085 to 1661520) | 886.61 (765.97 to 1028.05) | 3250568 (2659571 to 3886118) | 734.20 (603.69 to 879.98) | -0.608 (-0.63 to -0.592) | 20728 (12505 to 32285) | 13.58 (8.33 to 20.93) | 56784 (34786 to 84861) | 10.95 (6.54 to 16.92) | -0.695 (-0.707 to -0.683) |
| Jordan | 12601 (10706 to 14930) | 740.35 (632.58 to 873.56) | 50998 (43823 to 61359) | 628.10 (539.25 to 755.25) | -0.534 (-0.547 to -0.524) | 380 (252 to 554) | 23.02 (15.08 to 33.12) | 1410 (924 to 2118) | 17.22 (11.26 to 25.93) | -0.927 (-0.938 to -0.919) |
| Kazakhstan | 199284 (161897 to 240544) | 1583.05 (1295.79 to 1888.72) | 255971 (208477 to 311648) | 1518.23 (1257.09 to 1842.71) | -0.138 (-0.148 to -0.13) | 6377 (4193 to 9382) | 49.75 (32.77 to 72.34) | 6729 (4307 to 9896) | 38.54 (24.71 to 56.24) | -0.822 (-0.83 to -0.813) |
| Kenya | 40040 (36770 to 43815) | 460.77 (419.51 to 511.15) | 102742 (94500 to 112401) | 449.23 (411.00 to 491.45) | -0.086 (-0.101 to -0.073) | 1128 (745 to 1620) | 13.84 (9.14 to 19.64) | 2666 (1757 to 3862) | 12.55 (8.30 to 18.03) | -0.314 (-0.32 to -0.308) |
| Kiribati | 495 (424 to 577) | 1252.66 (1050.23 to 1484.87) | 1026 (854 to 1221) | 1359.33 (1116.96 to 1614.36) | 0.258 (0.253 to 0.264) | 16 (11 to 24) | 44.49 (29.11 to 65.45) | 30 (19 to 44) | 43.92 (28.21 to 64.21) | -0.048 (-0.053 to -0.043) |
| Kuwait | 5691 (5037 to 6437) | 657.68 (572.06 to 764.04) | 16238 (13652 to 19100) | 506.56 (432.95 to 619.24) | -0.848 (-0.868 to -0.833) | 119 (75 to 178) | 13.58 (8.64 to 20.61) | 348 (216 to 519) | 10.14 (6.33 to 15.04) | -0.942 (-0.957 to -0.928) |
| Kyrgyzstan | 50140 (41566 to 59729) | 1636.81 (1352.55 to 1959.55) | 71058 (59857 to 84198) | 1475.90 (1244.19 to 1752.72) | -0.334 (-0.338 to -0.33) | 1567 (1025 to 2242) | 50.64 (33.26 to 72.35) | 1944 (1270 to 2883) | 38.84 (25.40 to 57.49) | -0.854 (-0.863 to -0.846) |
| Lao People's Democratic Republic | 26136 (21905 to 30750) | 1225.38 (1015.52 to 1440.32) | 56353 (47046 to 67497) | 1212.79 (1013.98 to 1443.09) | -0.033 (-0.038 to -0.028) | 870 (586 to 1250) | 44.01 (29.64 to 63.32) | 1509 (960 to 2222) | 34.84 (22.36 to 51.54) | -0.751 (-0.76 to -0.744) |
| Latvia | 29199 (23816 to 36382) | 849.99 (700.81 to 1038.26) | 34864 (28045 to 43809) | 849.62 (698.46 to 1049.25) | 0.002 (-0.007 to 0.008) | 643 (412 to 956) | 19.06 (12.29 to 28.29) | 676 (403 to 1023) | 16.95 (10.33 to 25.72) | -0.379 (-0.382 to -0.375) |
| Lebanon | 14735 (12615 to 17908) | 684.29 (585.19 to 820.03) | 32829 (27790 to 39320) | 530.17 (448.51 to 633.40) | -0.821 (-0.826 to -0.816) | 400 (260 to 573) | 18.50 (12.03 to 26.56) | 787 (504 to 1147) | 12.80 (8.19 to 18.71) | -1.184 (-1.191 to -1.179) |
| Lesotho | 9701 (8209 to 11842) | 1170.68 (990.46 to 1445.33) | 12514 (10530 to 15285) | 1209.37 (1014.91 to 1480.63) | 0.131 (0.105 to 0.165) | 281 (182 to 416) | 33.61 (21.57 to 50.44) | 354 (227 to 514) | 34.07 (21.55 to 49.57) | 0.07 (0.044 to 0.106) |
| Liberia | 10857 (9102 to 12784) | 893.55 (754.26 to 1061.88) | 23539 (19841 to 28861) | 939.03 (794.85 to 1124.71) | 0.161 (0.154 to 0.168) | 492 (322 to 698) | 39.32 (25.91 to 55.52) | 946 (622 to 1348) | 35.34 (22.94 to 49.42) | -0.34 (-0.351 to -0.33) |
| Libya | 15909 (13604 to 18596) | 717.48 (600.91 to 861.99) | 34467 (29123 to 42090) | 624.81 (525.76 to 754.36) | -0.444 (-0.448 to -0.439) | 390 (244 to 583) | 17.57 (11.10 to 25.57) | 846 (545 to 1280) | 15.41 (9.89 to 22.91) | -0.417 (-0.434 to -0.402) |
| Lithuania | 37696 (30700 to 46144) | 853.49 (706.40 to 1034.86) | 49148 (39105 to 60981) | 824.73 (674.35 to 1006.91) | -0.109 (-0.114 to -0.104) | 829 (503 to 1301) | 19.12 (11.60 to 29.72) | 944 (587 to 1448) | 16.28 (10.11 to 24.82) | -0.514 (-0.528 to -0.504) |
| Luxembourg | 2755 (2216 to 3451) | 536.82 (438.99 to 656.07) | 4666 (3659 to 5726) | 396.50 (315.38 to 477.20) | -0.972 (-0.978 to -0.966) | 46 (28 to 71) | 9.02 (5.45 to 13.99) | 71 (42 to 111) | 5.92 (3.51 to 9.28) | -1.346 (-1.354 to -1.338) |
| Madagascar | 28986 (24386 to 33804) | 512.83 (425.93 to 605.85) | 65929 (55558 to 78474) | 517.21 (433.71 to 625.34) | 0.023 (0.01 to 0.034) | 1024 (680 to 1463) | 18.68 (12.46 to 26.74) | 1932 (1269 to 2897) | 16.25 (10.74 to 23.83) | -0.452 (-0.461 to -0.445) |
| Malawi | 26871 (23106 to 32076) | 613.25 (523.76 to 731.64) | 58641 (49652 to 67819) | 638.66 (529.30 to 754.26) | 0.137 (0.126 to 0.147) | 1081 (723 to 1503) | 23.78 (15.77 to 33.69) | 1614 (1005 to 2408) | 18.66 (11.92 to 27.30) | -0.77 (-0.78 to -0.759) |
| Malaysia | 93249 (83384 to 104303) | 968.68 (858.93 to 1098.80) | 326970 (271538 to 393666) | 1208.08 (1001.01 to 1456.34) | 0.726 (0.708 to 0.744) | 2838 (1892 to 4234) | 30.54 (20.42 to 45.61) | 7572 (4767 to 11272) | 29.04 (18.28 to 42.80) | -0.146 (-0.16 to -0.129) |
| Maldives | 1078 (893 to 1304) | 1218.34 (1002.08 to 1469.52) | 3294 (2721 to 3908) | 1045.34 (836.74 to 1274.99) | -0.491 (-0.5 to -0.483) | 34 (22 to 49) | 38.17 (24.51 to 54.20) | 83 (55 to 121) | 24.97 (16.19 to 36.20) | -1.362 (-1.376 to -1.35) |
| Mali | 44613 (38103 to 53529) | 1040.51 (895.47 to 1238.76) | 117887 (98122 to 137904) | 1136.44 (964.27 to 1339.87) | 0.286 (0.281 to 0.29) | 1955 (1308 to 2801) | 44.87 (30.08 to 64.37) | 4439 (3010 to 6197) | 42.15 (28.15 to 59.64) | -0.198 (-0.206 to -0.19) |
| Malta | 2300 (1870 to 2854) | 589.07 (479.47 to 724.08) | 4874 (3917 to 6074) | 456.81 (375.57 to 565.03) | -0.814 (-0.824 to -0.806) | 40 (25 to 62) | 10.67 (6.70 to 16.21) | 81 (50 to 121) | 7.41 (4.57 to 11.08) | -1.172 (-1.189 to -1.16) |
| Marshall Islands | 205 (174 to 245) | 1151.20 (953.75 to 1385.53) | 443 (364 to 530) | 1273.10 (1057.23 to 1522.55) | 0.324 (0.319 to 0.327) | 6 (4 to 9) | 36.96 (24.27 to 52.22) | 11 (7 to 17) | 37.77 (24.67 to 55.39) | 0.075 (0.067 to 0.082) |
| Mauritania | 11174 (9497 to 13081) | 1040.71 (887.79 to 1209.91) | 24164 (19924 to 29308) | 1033.32 (861.00 to 1241.92) | -0.021 (-0.026 to -0.016) | 407 (264 to 586) | 38.12 (25.33 to 54.93) | 680 (439 to 1015) | 29.22 (18.97 to 43.59) | -0.85 (-0.856 to -0.843) |
| Mauritius | 9507 (7674 to 11495) | 1321.30 (1067.59 to 1581.82) | 26266 (21625 to 31785) | 1499.83 (1258.75 to 1791.20) | 0.418 (0.404 to 0.434) | 237 (154 to 356) | 34.71 (22.70 to 52.41) | 608 (383 to 921) | 35.18 (22.45 to 53.61) | 0.056 (0.043 to 0.073) |
| Mexico | 301589 (279340 to 330425) | 739.99 (681.18 to 811.10) | 891775 (818690 to 976313) | 742.06 (677.41 to 813.05) | 0.008 (-0.001 to 0.017) | 6037 (3946 to 8930) | 14.87 (9.75 to 21.80) | 16352 (10459 to 24204) | 13.63 (8.76 to 20.06) | -0.284 (-0.296 to -0.273) |
| Micronesia (Federated States of) | 696 (585 to 820) | 1315.25 (1098.63 to 1567.76) | 1125 (934 to 1337) | 1516.28 (1263.61 to 1803.86) | 0.459 (0.449 to 0.467) | 21 (14 to 30) | 42.21 (27.25 to 60.79) | 28 (18 to 42) | 42.99 (27.53 to 62.95) | 0.057 (0.047 to 0.065) |
| Monaco | 285 (229 to 359) | 379.26 (309.60 to 466.48) | 401 (310 to 521) | 337.23 (274.77 to 420.35) | -0.377 (-0.381 to -0.373) | 4 (2 to 6) | 5.27 (3.07 to 8.38) | 5 (3 to 9) | 4.46 (2.68 to 7.32) | -0.534 (-0.543 to -0.526) |
| Mongolia | 15704 (13817 to 18205) | 1441.88 (1237.27 to 1705.44) | 27107 (23241 to 31521) | 1283.14 (1091.83 to 1506.27) | -0.378 (-0.382 to -0.374) | 595 (403 to 838) | 52.06 (34.96 to 74.01) | 828 (542 to 1193) | 36.25 (23.93 to 52.49) | -1.174 (-1.189 to -1.157) |
| Montenegro | 3900 (3266 to 4669) | 637.24 (534.81 to 755.31) | 5240 (4324 to 6511) | 634.19 (532.34 to 759.12) | -0.014 (-0.02 to -0.007) | 84 (55 to 126) | 13.99 (9.10 to 20.91) | 107 (69 to 164) | 13.00 (8.40 to 20.07) | -0.239 (-0.252 to -0.227) |
| Morocco | 93131 (80332 to 110442) | 579.28 (498.67 to 683.22) | 168297 (138325 to 202626) | 498.14 (413.11 to 592.20) | -0.478 (-0.487 to -0.47) | 2718 (1755 to 4007) | 17.08 (11.10 to 25.01) | 4365 (2825 to 6308) | 13.04 (8.40 to 18.84) | -0.858 (-0.866 to -0.85) |
| Mozambique | 28819 (24820 to 33622) | 458.38 (392.92 to 544.02) | 58035 (49299 to 67409) | 457.92 (378.84 to 549.60) | -0.006 (-0.019 to 0.005) | 1260 (854 to 1783) | 19.49 (13.17 to 27.95) | 2373 (1596 to 3379) | 17.99 (12.07 to 25.67) | -0.254 (-0.261 to -0.247) |
| Myanmar | 266070 (222707 to 316830) | 1135.89 (945.20 to 1362.80) | 519784 (426507 to 612352) | 1121.82 (917.91 to 1332.02) | -0.022 (-0.039 to -0.006) | 8904 (5995 to 12888) | 40.01 (26.95 to 58.03) | 14718 (9749 to 21618) | 32.79 (21.92 to 48.05) | -0.634 (-0.646 to -0.616) |
| Namibia | 9500 (8072 to 11011) | 1394.21 (1197.65 to 1624.24) | 16022 (13642 to 18802) | 1162.84 (984.07 to 1349.33) | -0.571 (-0.586 to -0.55) | 272 (170 to 395) | 39.75 (25.04 to 58.45) | 378 (237 to 562) | 27.93 (17.58 to 40.69) | -1.111 (-1.128 to -1.092) |
| Nauru | 66 (55 to 78) | 1331.02 (1121.11 to 1600.57) | 90 (75 to 107) | 1465.80 (1217.43 to 1756.07) | 0.314 (0.304 to 0.323) | 2 (1 to 3) | 39.45 (25.35 to 59.38) | 2 (1 to 3) | 40.52 (25.79 to 59.72) | 0.087 (0.074 to 0.101) |
| Nepal | 189394 (158303 to 224471) | 1832.39 (1541.71 to 2160.72) | 546232 (453993 to 669574) | 2298.92 (1904.13 to 2765.01) | 0.732 (0.719 to 0.746) | 9232 (6260 to 13063) | 91.19 (61.78 to 127.13) | 19588 (12474 to 28743) | 84.28 (54.64 to 123.86) | -0.264 (-0.281 to -0.249) |
| Netherlands | 87226 (72717 to 105755) | 440.40 (367.40 to 527.00) | 140146 (112994 to 171641) | 371.51 (305.00 to 445.53) | -0.548 (-0.558 to -0.538) | 1512 (919 to 2316) | 7.63 (4.67 to 11.64) | 2238 (1340 to 3479) | 5.83 (3.54 to 9.00) | -0.862 (-0.87 to -0.855) |
| New Zealand | 17639 (15123 to 20403) | 475.91 (406.90 to 551.05) | 37779 (31285 to 45827) | 418.26 (351.11 to 502.94) | -0.444 (-0.461 to -0.427) | 336 (206 to 504) | 9.24 (5.79 to 13.93) | 658 (389 to 1024) | 7.24 (4.31 to 11.29) | -0.805 (-0.826 to -0.785) |
| Nicaragua | 15914 (13913 to 18629) | 939.49 (806.16 to 1113.64) | 49290 (41752 to 58148) | 1016.32 (855.69 to 1209.13) | 0.253 (0.246 to 0.259) | 399 (257 to 597) | 24.79 (16.08 to 37.36) | 1106 (696 to 1634) | 23.39 (14.70 to 34.43) | -0.191 (-0.199 to -0.181) |
| Niger | 32146 (26847 to 37941) | 1009.48 (840.85 to 1212.68) | 94104 (78644 to 109967) | 1021.88 (846.05 to 1204.26) | 0.043 (0.036 to 0.048) | 1191 (793 to 1753) | 37.28 (24.55 to 53.76) | 2960 (1957 to 4430) | 33.43 (21.89 to 49.37) | -0.345 (-0.355 to -0.337) |
| Nigeria | 782690 (677604 to 909794) | 1660.90 (1421.82 to 1930.33) | 1769453 (1539190 to 1987103) | 1687.90 (1469.66 to 1904.23) | 0.053 (0.042 to 0.061) | 28633 (19190 to 40985) | 60.78 (40.58 to 86.83) | 59367 (39111 to 85442) | 56.22 (36.82 to 80.30) | -0.252 (-0.262 to -0.243) |
| Niue | 29 (24 to 35) | 1280.41 (1061.00 to 1532.11) | 29 (24 to 35) | 1390.13 (1158.23 to 1653.27) | 0.256 (0.246 to 0.264) | 1 (1 to 1) | 35.20 (22.27 to 51.62) | 1 (0 to 1) | 34.73 (22.29 to 50.50) | -0.053 (-0.06 to -0.046) |
| North Macedonia | 15453 (12993 to 18693) | 844.19 (715.34 to 1012.25) | 22735 (18915 to 27591) | 829.88 (702.40 to 984.42) | -0.061 (-0.071 to -0.051) | 412 (272 to 620) | 22.72 (15.13 to 33.85) | 519 (335 to 764) | 18.87 (12.13 to 27.29) | -0.599 (-0.609 to -0.588) |
| Northern Mariana Islands | 306 (252 to 371) | 1483.74 (1231.52 to 1788.62) | 732 (590 to 903) | 1509.23 (1244.96 to 1805.82) | 0.059 (0.053 to 0.065) | 6 (4 to 9) | 32.55 (19.81 to 49.28) | 14 (9 to 23) | 33.14 (20.75 to 51.44) | 0.062 (0.047 to 0.076) |
| Norway | 26766 (22191 to 32363) | 370.00 (308.82 to 439.21) | 36293 (29947 to 43786) | 313.96 (260.39 to 376.65) | -0.529 (-0.542 to -0.519) | 435 (262 to 680) | 5.92 (3.59 to 9.04) | 549 (323 to 870) | 4.63 (2.71 to 7.33) | -0.791 (-0.803 to -0.781) |
| Oman | 9183 (7802 to 10864) | 1126.33 (907.66 to 1358.62) | 20136 (16634 to 24507) | 882.04 (712.01 to 1112.22) | -0.783 (-0.793 to -0.773) | 182 (114 to 272) | 22.36 (13.96 to 33.37) | 335 (207 to 503) | 15.30 (9.36 to 23.59) | -1.214 (-1.222 to -1.206) |
| Pakistan | 569669 (499261 to 660746) | 972.56 (848.22 to 1131.25) | 1633816 (1430198 to 1872955) | 1203.47 (1055.16 to 1394.71) | 0.66 (0.614 to 0.686) | 28381 (19153 to 39585) | 48.39 (32.40 to 67.41) | 66500 (44813 to 94204) | 50.51 (34.21 to 72.53) | 0.101 (0.053 to 0.126) |
| Palau | 136 (114 to 160) | 1351.51 (1130.50 to 1600.69) | 309 (249 to 379) | 1466.18 (1215.65 to 1760.32) | 0.253 (0.245 to 0.261) | 3 (2 to 5) | 35.70 (22.61 to 51.83) | 7 (4 to 10) | 35.37 (22.57 to 53.36) | -0.035 (-0.044 to -0.027) |
| Palestine | 9170 (7825 to 10665) | 888.49 (758.23 to 1050.79) | 20109 (17064 to 24029) | 697.46 (591.34 to 831.45) | -0.779 (-0.785 to -0.774) | 232 (151 to 339) | 23.27 (15.04 to 33.38) | 441 (281 to 666) | 16.00 (10.16 to 24.07) | -1.198 (-1.205 to -1.193) |
| Panama | 11771 (10225 to 13594) | 744.48 (636.32 to 863.15) | 30670 (25935 to 35865) | 683.21 (577.54 to 800.12) | -0.274 (-0.28 to -0.269) | 251 (158 to 374) | 16.69 (10.55 to 24.58) | 616 (381 to 943) | 13.70 (8.49 to 21.06) | -0.641 (-0.651 to -0.629) |
| Papua New Guinea | 18657 (15898 to 21796) | 988.27 (837.20 to 1164.88) | 52591 (44624 to 61902) | 995.17 (838.74 to 1176.56) | 0.017 (0.012 to 0.022) | 543 (348 to 800) | 31.89 (21.41 to 45.45) | 1385 (896 to 2144) | 29.83 (19.47 to 44.90) | -0.217 (-0.228 to -0.207) |
| Paraguay | 21203 (17889 to 25224) | 943.65 (785.53 to 1135.82) | 46123 (39304 to 55245) | 801.91 (674.64 to 971.81) | -0.515 (-0.525 to -0.504) | 430 (272 to 647) | 19.06 (12.15 to 28.53) | 899 (560 to 1399) | 15.71 (9.76 to 24.30) | -0.614 (-0.624 to -0.603) |
| Peru | 79092 (68823 to 91579) | 570.38 (489.12 to 670.19) | 126022 (107881 to 146443) | 367.54 (312.34 to 429.23) | -1.41 (-1.426 to -1.394) | 2043 (1266 to 3003) | 14.70 (9.21 to 21.40) | 2691 (1707 to 3983) | 7.85 (4.96 to 11.65) | -2 (-2.015 to -1.986) |
| Philippines | 352568 (309274 to 402186) | 1162.59 (1017.00 to 1337.70) | 678417 (597102 to 770949) | 860.25 (756.53 to 981.38) | -0.974 (-0.991 to -0.957) | 9260 (6011 to 13649) | 33.37 (21.96 to 48.13) | 19974 (13065 to 28396) | 26.71 (17.62 to 37.60) | -0.714 (-0.723 to -0.706) |
| Poland | 312580 (265313 to 362612) | 782.92 (675.38 to 902.27) | 411439 (344844 to 485228) | 617.91 (525.66 to 725.46) | -0.769 (-0.775 to -0.761) | 7463 (4671 to 10870) | 18.91 (12.14 to 27.43) | 8485 (5294 to 12438) | 12.39 (7.69 to 18.16) | -1.356 (-1.368 to -1.344) |
| Portugal | 56155 (46085 to 68626) | 459.82 (382.59 to 548.92) | 88373 (69082 to 111230) | 309.83 (251.00 to 378.98) | -1.271 (-1.291 to -1.256) | 1043 (617 to 1594) | 8.82 (5.31 to 13.30) | 1580 (942 to 2463) | 5.34 (3.19 to 8.41) | -1.614 (-1.636 to -1.597) |
| Puerto Rico | 27249 (22034 to 34015) | 772.88 (629.32 to 965.24) | 46018 (35466 to 57711) | 622.29 (492.50 to 783.28) | -0.705 (-0.716 to -0.695) | 447 (269 to 700) | 13.25 (8.14 to 20.47) | 754 (435 to 1153) | 9.67 (5.71 to 15.00) | -1.012 (-1.018 to -1.006) |
| Qatar | 1567 (1285 to 1919) | 956.27 (778.58 to 1155.70) | 8388 (6978 to 10485) | 813.73 (643.58 to 1024.27) | -0.507 (-0.524 to -0.492) | 33 (20 to 50) | 22.18 (14.14 to 33.27) | 157 (97 to 240) | 15.84 (9.78 to 25.06) | -1.073 (-1.091 to -1.054) |
| Republic of Korea | 245246 (210038 to 284652) | 936.31 (779.10 to 1086.91) | 376465 (288157 to 460334) | 418.42 (317.74 to 512.50) | -2.569 (-2.597 to -2.552) | 3291 (1990 to 5192) | 14.59 (8.99 to 22.59) | 5202 (3146 to 8391) | 5.87 (3.52 to 9.54) | -2.892 (-2.908 to -2.878) |
| Republic of Moldova | 51872 (42536 to 64009) | 1284.10 (1059.50 to 1562.91) | 74372 (61177 to 90651) | 1272.25 (1056.82 to 1545.98) | -0.035 (-0.044 to -0.027) | 1353 (857 to 1989) | 33.20 (20.98 to 48.22) | 1747 (1089 to 2686) | 29.76 (18.75 to 45.72) | -0.36 (-0.369 to -0.351) |
| Romania | 183843 (151346 to 223463) | 750.75 (624.47 to 887.40) | 253928 (213032 to 308626) | 751.99 (634.85 to 923.90) | 0.005 (-0.014 to 0.024) | 4576 (2883 to 6708) | 19.15 (12.25 to 28.04) | 5338 (3382 to 8034) | 15.40 (9.81 to 22.90) | -0.702 (-0.719 to -0.684) |
| Russian Federation | 1309372 (1072347 to 1602765) | 801.47 (664.92 to 967.75) | 1573187 (1317357 to 1904227) | 681.31 (575.41 to 813.42) | -0.522 (-0.534 to -0.51) | 28827 (18069 to 43359) | 18.04 (11.39 to 26.99) | 30952 (19086 to 45822) | 13.54 (8.48 to 19.92) | -0.917 (-0.933 to -0.904) |
| Rwanda | 18631 (15780 to 22470) | 592.71 (501.33 to 719.62) | 33816 (27814 to 40772) | 516.51 (414.67 to 625.70) | -0.447 (-0.459 to -0.436) | 553 (358 to 813) | 19.05 (12.49 to 27.66) | 780 (492 to 1184) | 13.24 (8.34 to 19.69) | -1.171 (-1.181 to -1.161) |
| Saint Kitts and Nevis | 389 (321 to 474) | 1058.67 (889.11 to 1281.67) | 599 (478 to 745) | 914.58 (736.03 to 1126.28) | -0.485 (-0.498 to -0.474) | 8 (5 to 13) | 24.09 (15.50 to 35.67) | 10 (6 to 16) | 16.20 (9.84 to 25.75) | -1.269 (-1.283 to -1.255) |
| Saint Lucia | 864 (718 to 1032) | 959.59 (791.84 to 1145.87) | 2098 (1711 to 2556) | 903.61 (741.65 to 1092.21) | -0.201 (-0.213 to -0.19) | 18 (11 to 28) | 20.88 (13.41 to 31.93) | 39 (24 to 59) | 16.95 (10.72 to 25.90) | -0.672 (-0.681 to -0.665) |
| Saint Vincent and the Grenadines | 668 (556 to 801) | 908.23 (749.81 to 1091.95) | 1263 (1031 to 1555) | 930.36 (771.74 to 1135.98) | 0.072 (0.064 to 0.078) | 14 (9 to 21) | 19.47 (12.10 to 28.84) | 24 (15 to 36) | 18.07 (11.34 to 27.30) | -0.238 (-0.246 to -0.232) |
| Samoa | 918 (767 to 1105) | 1088.84 (902.51 to 1311.78) | 1603 (1335 to 1981) | 1155.50 (955.38 to 1428.40) | 0.194 (0.187 to 0.2) | 26 (17 to 40) | 33.42 (21.77 to 49.55) | 42 (27 to 63) | 32.37 (20.58 to 48.02) | -0.104 (-0.111 to -0.096) |
| San Marino | 137 (110 to 167) | 378.10 (308.40 to 458.35) | 344 (260 to 435) | 343.56 (277.07 to 418.73) | -0.309 (-0.315 to -0.303) | 2 (1 to 3) | 6.02 (3.63 to 9.35) | 5 (3 to 9) | 5.32 (3.18 to 8.36) | -0.394 (-0.406 to -0.385) |
| Sao Tome and Principe | 836 (703 to 982) | 1223.42 (1014.31 to 1439.85) | 1649 (1376 to 1955) | 1274.48 (1051.25 to 1520.37) | 0.134 (0.127 to 0.141) | 26 (17 to 38) | 38.10 (25.18 to 54.89) | 42 (27 to 61) | 33.48 (21.40 to 49.10) | -0.413 (-0.422 to -0.405) |
| Saudi Arabia | 68023 (58018 to 79226) | 1012.86 (837.50 to 1222.33) | 197667 (161692 to 235175) | 938.20 (755.04 to 1143.85) | -0.247 (-0.252 to -0.243) | 1743 (1119 to 2559) | 26.60 (17.01 to 40.13) | 4393 (2756 to 6683) | 21.04 (13.17 to 32.66) | -0.747 (-0.756 to -0.74) |
| Senegal | 27292 (23378 to 31653) | 770.43 (654.14 to 902.59) | 67084 (57837 to 78171) | 775.63 (661.74 to 905.93) | 0.027 (0.016 to 0.039) | 1207 (809 to 1710) | 32.67 (22.00 to 46.76) | 2298 (1519 to 3292) | 26.36 (17.59 to 37.74) | -0.687 (-0.698 to -0.675) |
| Serbia | 50073 (41175 to 62200) | 492.55 (410.35 to 593.94) | 65486 (53203 to 81162) | 430.29 (352.90 to 532.60) | -0.423 (-0.437 to -0.411) | 1081 (668 to 1619) | 11.24 (7.02 to 16.60) | 1308 (826 to 1958) | 8.25 (5.20 to 12.49) | -0.98 (-0.995 to -0.966) |
| Seychelles | 689 (568 to 817) | 1191.53 (978.91 to 1416.47) | 1344 (1097 to 1654) | 1235.75 (999.40 to 1506.39) | 0.127 (0.115 to 0.142) | 19 (12 to 27) | 32.23 (20.91 to 47.89) | 31 (20 to 47) | 29.33 (18.80 to 44.31) | -0.284 (-0.296 to -0.267) |
| Sierra Leone | 21283 (18298 to 24998) | 967.63 (827.17 to 1149.78) | 44310 (38116 to 51573) | 1036.37 (878.61 to 1230.97) | 0.219 (0.215 to 0.222) | 824 (555 to 1212) | 38.08 (25.28 to 55.19) | 1435 (945 to 2114) | 34.77 (23.06 to 51.02) | -0.294 (-0.304 to -0.285) |
| Singapore | 18432 (15304 to 21885) | 869.54 (716.26 to 1047.53) | 43940 (35089 to 57661) | 540.12 (431.34 to 706.09) | -1.516 (-1.525 to -1.505) | 264 (153 to 435) | 13.53 (8.13 to 21.64) | 567 (334 to 973) | 6.87 (4.12 to 11.72) | -2.145 (-2.16 to -2.13) |
| Slovakia | 36141 (30331 to 42565) | 662.47 (560.06 to 786.40) | 49407 (40558 to 60212) | 580.23 (483.86 to 701.61) | -0.42 (-0.427 to -0.412) | 828 (530 to 1242) | 15.26 (9.73 to 22.73) | 1006 (622 to 1534) | 11.52 (7.17 to 17.59) | -0.9 (-0.914 to -0.888) |
| Slovenia | 12062 (10083 to 14595) | 522.92 (438.11 to 621.68) | 20183 (16035 to 25551) | 442.41 (361.86 to 548.47) | -0.538 (-0.547 to -0.53) | 263 (166 to 393) | 11.54 (7.28 to 17.28) | 378 (227 to 589) | 8.25 (5.04 to 12.69) | -1.077 (-1.095 to -1.061) |
| Solomon Islands | 1885 (1586 to 2236) | 1234.12 (1050.49 to 1455.48) | 4913 (4055 to 5822) | 1295.61 (1072.66 to 1522.05) | 0.16 (0.156 to 0.164) | 56 (37 to 79) | 41.08 (27.39 to 59.08) | 131 (83 to 192) | 38.65 (24.74 to 55.71) | -0.195 (-0.204 to -0.187) |
| Somalia | 19869 (16698 to 23477) | 618.38 (525.04 to 723.64) | 51066 (43693 to 59222) | 615.51 (520.49 to 729.18) | -0.014 (-0.019 to -0.01) | 706 (467 to 1026) | 21.64 (14.18 to 31.27) | 1496 (942 to 2231) | 19.30 (12.28 to 28.18) | -0.365 (-0.374 to -0.358) |
| South Africa | 243861 (208920 to 284035) | 1145.24 (972.59 to 1348.63) | 486820 (431886 to 553515) | 1089.08 (963.50 to 1236.36) | -0.147 (-0.163 to -0.123) | 7093 (4650 to 10393) | 32.02 (21.02 to 46.89) | 13326 (8655 to 19348) | 28.77 (18.69 to 41.87) | -0.337 (-0.352 to -0.321) |
| South Sudan | 17440 (14782 to 20667) | 590.69 (499.96 to 709.55) | 26303 (22075 to 30701) | 572.92 (483.34 to 677.35) | -0.103 (-0.12 to -0.089) | 506 (333 to 731) | 17.97 (11.86 to 26.05) | 697 (440 to 1041) | 16.01 (10.20 to 23.97) | -0.377 (-0.387 to -0.368) |
| Spain | 309114 (249206 to 369083) | 590.31 (479.07 to 704.80) | 467780 (362779 to 595040) | 386.13 (309.95 to 476.83) | -1.351 (-1.362 to -1.341) | 4979 (3139 to 7648) | 9.61 (6.07 to 14.74) | 7337 (4383 to 11404) | 5.91 (3.60 to 9.29) | -1.546 (-1.561 to -1.533) |
| Sri Lanka | 127884 (107265 to 149951) | 1195.90 (988.82 to 1412.34) | 274071 (217783 to 342934) | 1071.31 (865.45 to 1319.76) | -0.349 (-0.36 to -0.339) | 3536 (2323 to 5291) | 33.97 (22.19 to 50.21) | 6398 (3952 to 9722) | 25.73 (16.14 to 38.93) | -0.892 (-0.901 to -0.884) |
| Sudan | 87061 (71189 to 104827) | 867.40 (712.72 to 1042.82) | 174390 (145625 to 206225) | 808.06 (674.71 to 964.77) | -0.225 (-0.233 to -0.218) | 2926 (1936 to 4197) | 29.51 (19.22 to 42.13) | 5103 (3295 to 7536) | 24.32 (15.55 to 35.42) | -0.62 (-0.627 to -0.613) |
| Suriname | 2657 (2228 to 3198) | 998.36 (824.98 to 1198.60) | 6217 (5126 to 7514) | 1008.41 (840.93 to 1213.33) | 0.025 (0.014 to 0.035) | 61 (39 to 91) | 22.68 (14.35 to 34.01) | 126 (80 to 192) | 20.69 (13.22 to 31.16) | -0.3 (-0.309 to -0.291) |
| Sweden | 58910 (48340 to 74024) | 375.84 (314.30 to 463.79) | 81834 (66067 to 102277) | 311.41 (258.06 to 380.23) | -0.599 (-0.612 to -0.588) | 993 (613 to 1518) | 6.21 (3.83 to 9.52) | 1270 (760 to 2027) | 4.74 (2.87 to 7.52) | -0.859 (-0.87 to -0.85) |
| Switzerland | 59094 (49861 to 71107) | 567.18 (482.76 to 677.12) | 95829 (77137 to 120169) | 464.50 (390.08 to 563.58) | -0.628 (-0.647 to -0.606) | 929 (586 to 1472) | 8.61 (5.33 to 13.63) | 1471 (887 to 2345) | 6.75 (4.12 to 10.59) | -0.751 (-0.777 to -0.723) |
| Syrian Arab Republic | 60796 (51672 to 71234) | 986.57 (834.23 to 1171.28) | 97370 (80814 to 118377) | 797.52 (666.08 to 970.03) | -0.679 (-0.694 to -0.667) | 1770 (1152 to 2645) | 28.65 (18.79 to 42.50) | 2496 (1577 to 3751) | 20.51 (13.27 to 30.67) | -1.07 (-1.085 to -1.057) |
| Taiwan (Province of China) | 117565 (98429 to 142559) | 818.44 (694.18 to 980.15) | 274579 (230669 to 337553) | 646.15 (541.45 to 787.11) | -0.758 (-0.8 to -0.723) | 2787 (1774 to 4177) | 21.39 (13.54 to 32.09) | 6217 (3840 to 9403) | 14.29 (8.93 to 21.70) | -1.317 (-1.373 to -1.273) |
| Tajikistan | 35546 (29926 to 41856) | 1266.27 (1062.29 to 1502.19) | 75033 (63852 to 89132) | 1330.72 (1109.32 to 1572.42) | 0.155 (0.145 to 0.164) | 1187 (792 to 1691) | 41.37 (27.98 to 59.09) | 2415 (1575 to 3453) | 40.65 (26.55 to 58.44) | -0.058 (-0.074 to -0.045) |
| Thailand | 508199 (434075 to 592000) | 1527.44 (1302.60 to 1808.25) | 1715726 (1387505 to 2056540) | 1607.78 (1297.76 to 1918.89) | 0.166 (0.147 to 0.182) | 12072 (7805 to 17674) | 38.24 (24.78 to 56.48) | 37253 (23542 to 55485) | 34.72 (21.89 to 51.58) | -0.309 (-0.319 to -0.299) |
| Timor-Leste | 3443 (2892 to 4112) | 1137.34 (942.77 to 1364.65) | 9371 (7602 to 11113) | 1118.13 (926.80 to 1321.97) | -0.048 (-0.06 to -0.032) | 91 (59 to 133) | 34.25 (22.29 to 48.95) | 226 (144 to 334) | 28.98 (18.74 to 43.22) | -0.531 (-0.545 to -0.517) |
| Togo | 15057 (12776 to 17670) | 1021.89 (851.17 to 1215.39) | 47634 (39837 to 55741) | 1097.46 (922.67 to 1311.14) | 0.234 (0.228 to 0.24) | 558 (360 to 815) | 38.47 (24.89 to 55.81) | 1611 (1051 to 2283) | 38.21 (24.74 to 54.50) | -0.015 (-0.025 to -0.004) |
| Tokelau | 16 (13 to 18) | 1199.94 (984.57 to 1408.50) | 19 (15 to 23) | 1269.68 (1029.06 to 1526.54) | 0.182 (0.175 to 0.188) | 0 (0 to 1) | 36.26 (23.04 to 53.04) | 0 (0 to 1) | 32.78 (20.67 to 46.78) | -0.324 (-0.331 to -0.318) |
| Tonga | 677 (550 to 803) | 1219.27 (998.75 to 1428.94) | 1047 (863 to 1238) | 1294.91 (1064.78 to 1545.76) | 0.191 (0.184 to 0.197) | 18 (11 to 26) | 34.00 (21.80 to 49.44) | 26 (17 to 39) | 33.41 (21.66 to 49.87) | -0.058 (-0.066 to -0.05) |
| Trinidad and Tobago | 7746 (6376 to 9399) | 911.39 (749.59 to 1110.88) | 16206 (13085 to 20028) | 883.87 (726.62 to 1074.47) | -0.108 (-0.122 to -0.095) | 154 (96 to 233) | 19.05 (11.83 to 28.73) | 284 (179 to 449) | 15.75 (9.86 to 24.73) | -0.615 (-0.623 to -0.607) |
| Tunisia | 30698 (26622 to 35907) | 605.95 (527.83 to 709.28) | 71319 (59158 to 85074) | 571.30 (478.91 to 677.45) | -0.185 (-0.192 to -0.176) | 890 (595 to 1270) | 17.97 (12.11 to 25.36) | 1901 (1237 to 2761) | 15.24 (9.87 to 22.18) | -0.515 (-0.527 to -0.505) |
| Turkmenistan | 29128 (24965 to 33947) | 1482.75 (1259.98 to 1746.10) | 60164 (50427 to 72596) | 1534.96 (1286.38 to 1857.24) | 0.111 (0.107 to 0.114) | 925 (612 to 1338) | 44.91 (29.77 to 65.39) | 1580 (1021 to 2294) | 38.46 (24.75 to 55.73) | -0.502 (-0.508 to -0.498) |
| Tuvalu | 80 (67 to 97) | 1238.67 (1038.02 to 1474.63) | 136 (114 to 162) | 1337.23 (1134.55 to 1591.84) | 0.246 (0.241 to 0.25) | 2 (2 to 4) | 40.85 (26.35 to 59.42) | 3 (2 to 5) | 36.74 (23.03 to 55.38) | -0.341 (-0.347 to -0.335) |
| Turkey | 297790 (258334 to 346485) | 778.82 (673.76 to 902.34) | 553692 (473933 to 652767) | 619.69 (528.58 to 728.95) | -0.717 (-0.737 to -0.691) | 8599 (5708 to 12565) | 23.27 (15.37 to 33.52) | 13910 (9039 to 20337) | 15.59 (10.15 to 22.92) | -1.257 (-1.284 to -1.235) |
| Uganda | 43413 (36838 to 50778) | 602.48 (502.54 to 712.99) | 100896 (86707 to 118102) | 585.27 (494.77 to 700.22) | -0.097 (-0.109 to -0.086) | 1518 (1000 to 2169) | 21.99 (14.53 to 31.11) | 2911 (1899 to 4333) | 17.84 (11.53 to 26.28) | -0.669 (-0.675 to -0.663) |
| Ukraine | 479930 (397236 to 600466) | 745.23 (616.24 to 917.06) | 577411 (476768 to 713237) | 761.20 (632.96 to 934.43) | 0.071 (0.06 to 0.08) | 11032 (6985 to 16351) | 17.33 (11.16 to 25.66) | 12458 (7814 to 18667) | 16.68 (10.45 to 24.99) | -0.118 (-0.142 to -0.101) |
| United Arab Emirates | 9768 (8191 to 11858) | 1290.71 (1067.34 to 1561.65) | 59631 (46548 to 78534) | 941.35 (770.23 to 1150.33) | -1.009 (-1.025 to -0.996) | 204 (122 to 307) | 27.81 (16.97 to 42.68) | 1114 (677 to 1787) | 19.45 (12.07 to 30.03) | -1.144 (-1.16 to -1.129) |
| United Kingdom | 495894 (415912 to 595000) | 557.37 (473.16 to 653.25) | 616841 (515475 to 731462) | 441.78 (372.13 to 520.13) | -0.745 (-0.752 to -0.738) | 8408 (5207 to 12581) | 9.41 (5.86 to 14.38) | 9957 (5868 to 15531) | 6.92 (4.11 to 10.85) | -0.986 (-0.999 to -0.974) |
| United Republic of Tanzania | 62830 (53352 to 73915) | 505.06 (428.67 to 593.74) | 161090 (136475 to 186203) | 521.20 (437.51 to 613.42) | 0.079 (0.041 to 0.114) | 2488 (1655 to 3540) | 19.22 (12.69 to 27.77) | 4508 (2865 to 6653) | 15.13 (9.83 to 22.21) | -0.794 (-0.829 to -0.763) |
| United States of America | 2014362 (1815307 to 2249758) | 616.66 (558.19 to 686.32) | 4349268 (3694305 to 5177287) | 729.43 (619.87 to 861.63) | 0.544 (0.523 to 0.562) | 33706 (21399 to 49574) | 10.23 (6.50 to 15.12) | 70394 (44252 to 107910) | 11.75 (7.37 to 17.92) | 0.454 (0.435 to 0.47) |
| United States Virgin Islands | 656 (525 to 808) | 813.81 (657.46 to 997.24) | 1295 (1045 to 1623) | 769.35 (637.05 to 924.69) | -0.18 (-0.184 to -0.176) | 11 (7 to 17) | 14.16 (8.43 to 21.84) | 20 (12 to 31) | 12.33 (7.58 to 19.46) | -0.438 (-0.447 to -0.429) |
| Uruguay | 19539 (16001 to 24103) | 524.12 (432.23 to 635.33) | 25043 (20390 to 31233) | 428.25 (352.08 to 538.43) | -0.655 (-0.673 to -0.637) | 364 (224 to 555) | 9.81 (6.08 to 15.08) | 484 (297 to 734) | 7.50 (4.60 to 11.49) | -0.888 (-0.907 to -0.866) |
| Uzbekistan | 228549 (192360 to 278051) | 1820.95 (1529.03 to 2222.88) | 481418 (398055 to 589799) | 1834.86 (1542.38 to 2205.31) | 0.021 (0.016 to 0.027) | 8062 (5295 to 11853) | 62.49 (40.87 to 92.31) | 15245 (9673 to 23180) | 54.75 (34.88 to 82.32) | -0.432 (-0.441 to -0.424) |
| Vanuatu | 781 (664 to 924) | 1175.51 (983.31 to 1403.48) | 2430 (2041 to 2858) | 1336.44 (1122.25 to 1580.89) | 0.416 (0.409 to 0.422) | 22 (14 to 32) | 36.76 (23.88 to 53.14) | 65 (41 to 96) | 40.04 (25.48 to 56.83) | 0.275 (0.267 to 0.282) |
| Venezuela (Bolivarian Republic of) | 72338 (61962 to 83586) | 713.87 (602.03 to 840.55) | 196762 (166985 to 233529) | 684.43 (579.85 to 815.68) | -0.136 (-0.147 to -0.127) | 1692 (1062 to 2559) | 17.71 (11.26 to 26.76) | 4424 (2861 to 6671) | 15.64 (10.10 to 23.58) | -0.381 (-0.4 to -0.362) |
| Viet Nam | 276650 (232220 to 332031) | 683.78 (571.05 to 823.88) | 480821 (397959 to 584406) | 534.65 (439.97 to 653.74) | -0.774 (-0.79 to -0.757) | 8374 (5336 to 12523) | 21.37 (13.69 to 31.65) | 12762 (8139 to 19083) | 14.63 (9.38 to 21.84) | -1.208 (-1.221 to -1.192) |
| Yemen | 32981 (27391 to 39113) | 700.65 (566.78 to 839.30) | 93178 (79480 to 111114) | 637.61 (532.26 to 767.42) | -0.302 (-0.307 to -0.296) | 1506 (1030 to 2129) | 29.08 (19.66 to 41.35) | 4099 (2857 to 5856) | 26.61 (18.03 to 38.09) | -0.279 (-0.289 to -0.272) |
| Zambia | 20258 (17075 to 23465) | 610.98 (504.83 to 723.87) | 60493 (50392 to 70280) | 704.34 (582.36 to 833.44) | 0.464 (0.451 to 0.477) | 769 (521 to 1106) | 22.76 (15.28 to 32.93) | 1782 (1157 to 2668) | 21.30 (13.90 to 31.26) | -0.204 (-0.217 to -0.193) |
| Zimbabwe | 55126 (46156 to 65752) | 1276.44 (1076.38 to 1502.79) | 85334 (71255 to 100877) | 1215.38 (1014.43 to 1450.00) | -0.136 (-0.15 to -0.115) | 1630 (1054 to 2372) | 37.16 (24.37 to 54.66) | 2677 (1752 to 3849) | 37.58 (25.31 to 53.97) | 0.067 (0.038 to 0.097) |

AAPC, average annual percentage change; CI, confidence interval; CKD, chronic kidney disease; UI, uncertainty interval; YLDs, years lost due to disability.

Table S2 The predictions of prevalence and YLDs of anemia attributed to CKD from 2022 to 2030.

| Year | Prevalence | | YLDs | |
| --- | --- | --- | --- | --- |
|  | cases (95% UI) | rate per 100,000 (95% UI) | cases (95% UI) | rate per 100,000 (95% UI) |
| 2022 | 61928838 (60856543 to 63001133) | 769.74 (756.42 to 783.07) | 1638611 (1614538 to 1662684) | 20.37 (20.07 to 20.66) |
| 2023 | 62219174 (60847632 to 63590715) | 765.98 (749.1 to 782.87) | 1639963 (1609805 to 1670121) | 20.19 (19.82 to 20.56) |
| 2024 | 62487842 (60855575 to 64120108) | 762.16 (742.25 to 782.07) | 1640899 (1605449 to 1676349) | 20.01 (19.58 to 20.45) |
| 2025 | 62735950 (60865203 to 64606698) | 758.31 (735.7 to 780.93) | 1641527 (1601259 to 1681796) | 19.84 (19.36 to 20.33) |
| 2026 | 62955401 (60860656 to 65050147) | 754.35 (729.25 to 779.45) | 1641419 (1596640 to 1686198) | 19.67 (19.13 to 20.2) |
| 2027 | 63145781 (60836828 to 65454734) | 750.25 (722.82 to 777.69) | 1640352 (1591264 to 1689439) | 19.49 (18.91 to 20.07) |
| 2028 | 63312074 (60797332 to 65826816) | 746.09 (716.46 to 775.73) | 1638591 (1585401 to 1691781) | 19.31 (18.68 to 19.94) |
| 2029 | 63459893 (60746228 to 66173558) | 741.93 (710.21 to 773.66) | 1636496 (1579380 to 1693612) | 19.13 (18.47 to 19.8) |
| 2030 | 63590459 (60683343 to 66497575) | 737.79 (704.06 to 771.52) | 1634167 (1573263 to 1695071) | 18.96 (18.25 to 19.67) |
| 2031 | 63696452 (60600523 to 66792381) | 733.57 (697.92 to 769.23) | 1631267 (1566686 to 1695849) | 18.79 (18.04 to 19.53) |
| 2032 | 63776614 (60495413 to 67057815) | 729.27 (691.75 to 766.79) | 1627569 (1559389 to 1695749) | 18.61 (17.83 to 19.39) |
| 2033 | 63838474 (60375713 to 67301234) | 724.97 (685.65 to 764.29) | 1623380 (1551706 to 1695055) | 18.44 (17.62 to 19.25) |
| 2034 | 63887435 (60246470 to 67528400) | 720.72 (679.65 to 761.8) | 1619020 (1543947 to 1694094) | 18.26 (17.42 to 19.11) |
| 2035 | 63922645 (60106480 to 67738811) | 716.52 (673.75 to 759.3) | 1614511 (1536119 to 1692903) | 18.1 (17.22 to 18.98) |

UI, uncertainty interval; CKD, chronic kidney disease; YLDs, years lost due to disability.


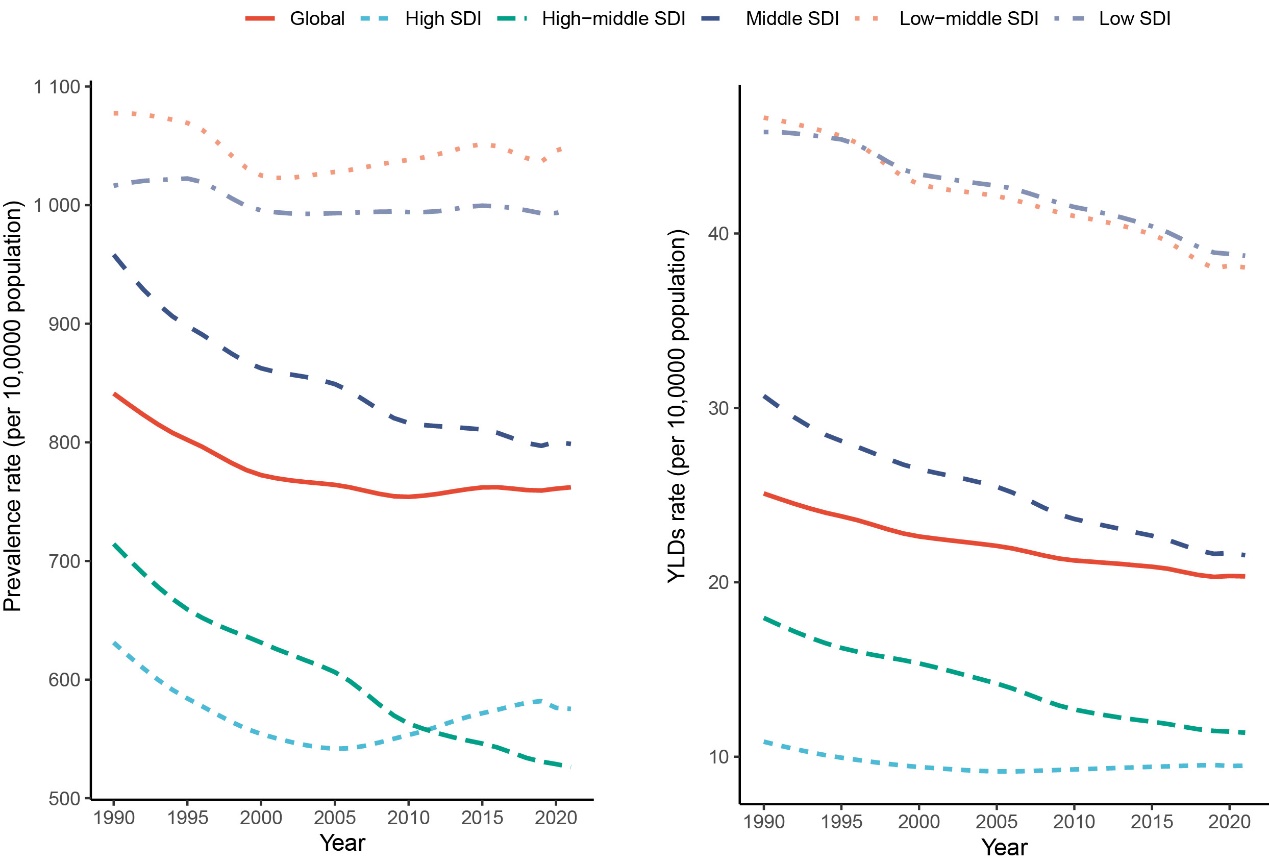


Figure S1. Global anemia burden attributed to CKD by SDI quintiles from 1990 to 2021. (A) age-standardized prevalence rate; (B) age-standardized YLDs rate. CKD, chronic kidney disease; SDI, socio-demographic index; YLDs, years lost due to disability.
